# Identification, analysis of deleterious SNPs of the human *GSR* gene and their effects on the structure and functions of associated proteins and other diseases

**DOI:** 10.1038/s41598-022-09295-6

**Published:** 2022-03-31

**Authors:** Bharti Vyas, Ratul Bhowmik, Mymoona Akhter, Farhan Jalees Ahmad

**Affiliations:** 1 School of Interdisciplinary Studies, Jamia Hamdard, New Delhi, India; 2 Department of Pharmaceutical Chemistry, School of Pharmaceutical Education and Research, Jamia Hamdard, New Delhi, 110062 India

**Keywords:** Computational biology and bioinformatics, Biomarkers, Medical research, Risk factors

## Abstract

Hereditary glutathione reductase deficiency, caused by mutations of the *GSR* gene, is an autosomal recessive disorder characterized by decreased glutathione disulfide (GSSG) reduction activity and increased thermal instability. This study implemented computational analysis to screen the most likely mutation that might be associated with hereditary glutathione reductase deficiency and other diseases. Using ten online computational tools, the study revealed four nsSNPs among the 17 nsSNPs identified as most deleterious and disease associated. Structural analyses and evolutionary confirmation study of native and mutant GSR proteins using the HOPE project and ConSruf. HOPE revealed more flexibility in the native *GSR* structure than in the mutant structure. The mutation in *GSR* might be responsible for changes in the structural conformation and function of the GSR protein and might also play a significant role in inducing hereditary glutathione reductase deficiency. LD and haplotype studies of the gene revealed that the identified variations rs2978663 and rs8190955 may be responsible for obstructive heart defects (OHDs) and hereditary anemia, respectively. These interethnic differences in the frequencies of SNPs and haplotypes might help explain the unpredictability that has been reported in association studies and can contribute to predicting the pharmacokinetics and pharmacodynamics of drugs that make use of *GSR*.

## Introduction

Glutathione-disulfide reductase (GSR) protein is also known as Glutathione reductase (GR) enzyme encoded by the *GSR* gene in humans, which is located on chromosome 8p21 and consists of 13 exons^1–3^. Glutathione-disulfide reductase is a 522-amino acid protein that initiates the synthesis of mitochondrial and cytosolic GR. It is a member of the class-1 pyridine nucleotide-disulfide oxidoreductase family. Glutathione reductase is a homodimeric flavoprotein, a central enzyme of cellular antioxidant defense^4^, and reduces oxidized glutathione disulfide (GSSG) to the sulfhydryl form GSH, which works as a cellular antioxidant^5,6^.

Glutathione reductase catalyses the reaction (GSSG + NADPH + H + 2 (GSH) + NADP +) which is an important enzyme in this cellular system. Because it maintains the ratio of GSH/GSSG, it is involved in many cellular functions, including the activation of dormant cells^7,8^. GSH plays a key role in two ways: one maintaining the function and preventing oxidative stress in red blood cells^4^ and second clearing the electrophilic xenobiotics.

Hereditary glutathione reductase deficiency generally impairs cellular energy balance and increases the level of oxidative stress in red blood cells and has been related to hereditary hemolytic anemia^9,10^.

Familial deficiency of glutathione reductase in human blood cells has been reported by Loose et. al. and Roos et. al. in 1976 and 1979 respectively indicating the importance of GSH^11,12^. Non-synonymous single nucleotide polymorphisms (nsSNPs) occurring in the coding regions of gene that result in point mutations affect protein function and lead to pathogenic phenotypes^13,14^. nsSNP have the potential to alter the function of their protein, either directly or via disruption of structure^15^. To date, 18 mutations are reported in *GSR* gene which is responsible for glutathione reductase deficiency^16–18^. Mutations in *GSR* gene were reported in a northern Thailand population^19^ for the first time and subsequently in African Americans^20^, and Korea^21^ populations. It has been reported that the mutations in *GSR* gene cause glutathione reductase deficiency leading to reduced lifespan of red blood cells (RBCs)^12^.

Computational techniques has proven to be beneficial in determining the mutation and related effects effectively like finding out the non-significant SNPs from the significant SNPs that might produce more deleterious disease-associated consequences^22^. Incorporating the phenotypic changes along with the classical computational SNP prediction techniques have provided a high accuracy prediction level and helped in the classification of SNPs on the basis of their specific disease-associated consequences. This study examined and reports the most deleterious effect of nsSNPs reported in the Glutathione-disulfide reductase protein coding region. During this study SIFT^23^, Polyphen^24^, Mutation testers^31^, SNPs&Go^28^, SNAP2^29^, and Provean tools^30^ were used to prioritize the deleterious disease-associated nsSNPs from the available SNP datasets obtained from the ClinVar^25^ database. In addition, a linkage disequilibrium (LD) and haplotype-based approach were used to examine *GSR* genes for linkage and association with other diseases. A high-resolution haplotype structure for the *GSR* gene and the association of the individual SNPs and haplotypes with the *GSR* gene in Han Chinese (CHB) individuals from the International Hapmap Project has been defined. The deleterious polymorphisms of gene and their impact on function of protein, association and linkage of selected gene polymorphisms of the glutathione S-reductase (*GSR*) with various defects/disorders like Obstructive Heart Defects (OHDs)) and hereditary anaemia in humans is also presented here. The findings of this study would be helpful in the development of personalized medicine.

## Result

### Mutation spread of *GSR* gene

**Figure 1:**
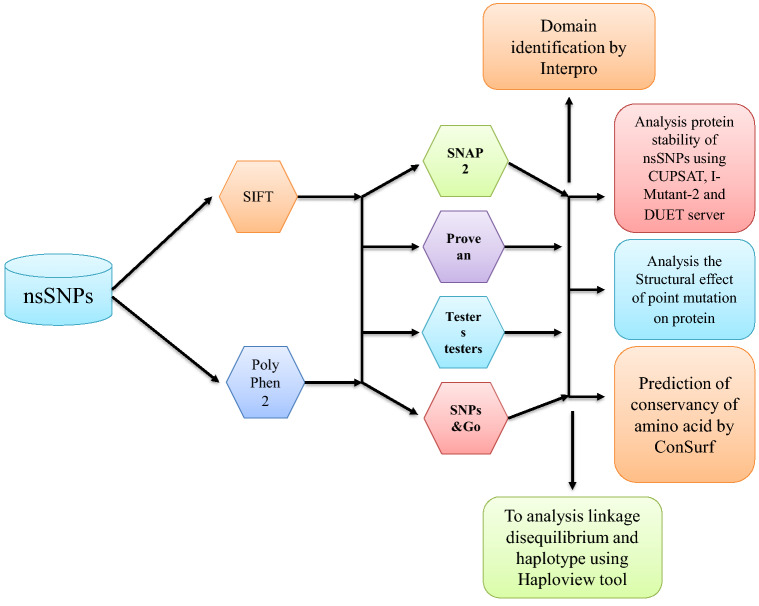
Schematic diagram summarizing the study.

The SNPs present in the *GSR* gene were retrieved using the ClinVar database, and 18 various mutations were found in the gene. Among the coding regions of mutations identified, 17 were related to missense mutations, and 1 was related to nonsense mutations. An overview of the complete methodological approaches is summarized in a schematic diagram (Fig. 1).

### Identification of deleterious missense mutation

Six pathogenicity prediction web servers were used to predict the deleterious effect of nsSNPs. Six variants were observed to be damaged by using the SIFT tool and were further subjected to cross check by using five different tools (WHESS.db submodule PolyPhen-2 server, Mutation testers, SNPs&Go, SNAP2 and Provean tools). Out of a total of 17 nsSNPs identified, only four were predicted to be the most deleterious nsSNPs in all computational algorithms. The results are presented in Table 1.

**Table 1:** The list of deleterious nsSNPs predicted by SIFT, SNAP2, POLYPHEN, SNP&GO, Mutation tester, Provean and SNAP2 tools.

Tool	Parameter nsSNPs	Amino acid substitution (nsSNP ID)			
		V289A rs151187899	R233C rs145851500	A199T rs141805635	R153C rs8190955
SIFT prediction	Score prediction	0 Damaging	0 Damaging	0 Damaging	0 Damaging
POLYPHEN prediction	Score prediction	0.882 Probably damaging	1 Probably damaging	1 Probably damaging	0.932 Probably damaging
SNP&GO	Score prediction	0.837 Disease	0.801 Disease	0.873 Disease	0.524 Disease
Mutation tester	Score prediction	1 Disease causing	0.999 Disease causing	0.999 Disease causing	0.999 Disease causing
SNAP2	Score prediction	76 Effect	54 Effect	31 Effect	27 Effect
PROVEAN	Score prediction	−6.982 Deleterious	−3.733 Deleterious	−5.830 Deleterious	−9.026 Deleterious

### Identification of nsSNPs on the domains of proteins

InterPro, a domain identification tool, predicts the domains and active sites of a protein through the functional analysis of protein families. It predicted four functional domains of GSR, which are pyridine nucleotide-disulfide oxidoreductase domains and FAD/NAD(P)-binding domains (65–390), and demonstrated that all 4 nsSNPs identified are positioned on these domains (Fig. S1).

### Prediction of stability of the mutant protein

Protein stability was analysed by using the CUPSAT, I-Mutant-2 and DUET servers. The results revealed that four variants destabilized the *GSR* residue, namely, (V289A) rs151187899, (R233C) rs145851500, (A199T) rs141805635, and (R153C) rs8190955. The results are presented in Table 2.

**Table 2:** List of damaged nsSNPs and affected amino acids their prediction, DDG values after mutation by using I-Mutant, CUPSAT and DUET.

Gene name	nsSNP ID	Amino acid	CUPSAT		DUET		I-Mutant2
			Prediction	DDG value (Kcal/mol)	Prediction	DDG value (Kcal/mol)	DDG value (Kcal/mol)
GSR	rs151187899	V289A	Destabilizing	−5.4	Destabilizing	−0.68	−0.68
	rs145851500	R233C	Destabilizing	−0.13	Destabilizing	−1.352	−1.52
	rs141805635	A199T	Destabilizing	−2.65	Destabilizing	−1.621	−1.21
	rs8190955	R153C	Destabilizing	−0.11	Destabilizing	−2.869	−1.869

### Structural effect of point mutation on human GSR protein

Point mutation in genes has been studied by the Project HOPE server and the results revealed that the substitution of wild residues of R153C, R233C, A199T and V289A. These substitutions might be affecting the structure and function of the protein as the structure and physicochemical properties of different amino acids is diverse. For example in this case arginine residue is strong basic positively charged amino acid and has ability to form multiple hydrogen bonds and salt bridge in comparison to cysteine. Other mutations might be affecting the function and stability of the protein similarly (Table S2).

### Evolutionary conservation analysis

The evolutionary conservancy of amino acid residues of the native GSR was examined by the ConSurf web server. It identified structural and functional residues of the 4 high-risk nsSNPs of the GSR protein using evolutionary conservation and solvent accessibility. We observed that R153 and V289 are exposed and functional, whereas residues A199 and R233 are buried and structural. All 2 of these residues are highly conserved (Table S1).

### Study of LD and haplotype

LD (linkage disequilibrium) and haplotype were used to analyse the various genetic parameters of the *GSR* gene, and the genotype data of CHB (Han Chinese) were retrieved from the International Hapmap Project. Haplotype block reveals the combination of alleles at neighboring loci on the chromosome that may be transmitted together, and LD provides information on the measurement of the involvement of alleles (genetic marker) in a nonrandom mode.

**Figure 2:**
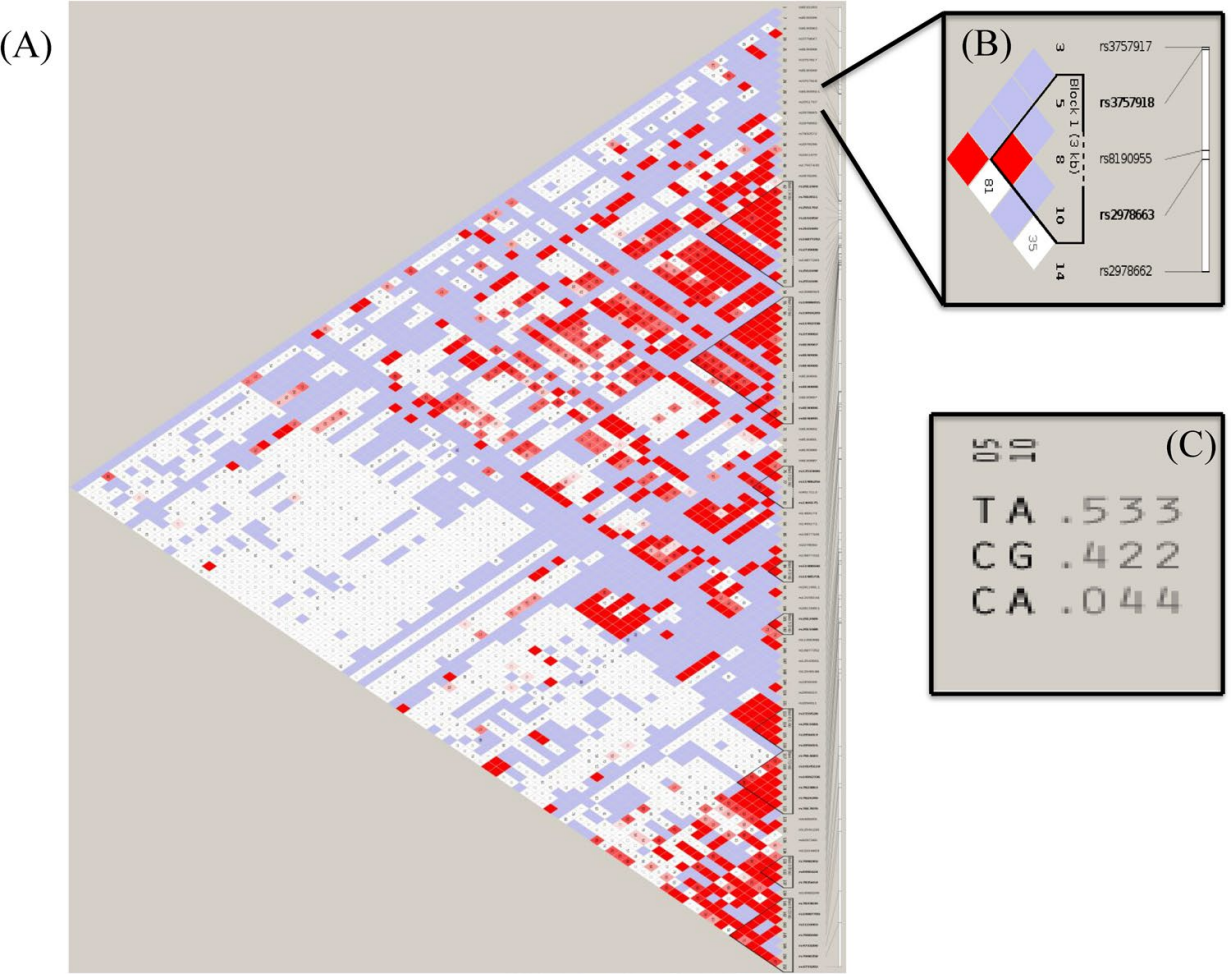
LD structure of GRS gene : **(A)** LD structure of maximum number of SNPs, **(B)** LD structure of GSR with minimum block size. **(C)** Haplotype block on GSR.

Information about the predisposition of various diseases due to genetic variants has been evaluated. These parameters work as vital biomarkers for functional associations with a variety of diseases. The LD and haplotype study revealed an important block in the *GSR* gene, with three important SNPs having nonrandom associations, as represented in Fig. 2. Three critical SNPs out of five SNPs identified were rs3757918, rs8190955 and rs2978663 with minor allele frequencies T:C, C:T and A:G, respectively, with r2 ≥ 0.8, showing a high correlation between the loci (Table S3).

Furthermore, one of the haplotype blocks generated of the *GSR* gene involving 2 SNPs revealed that these variations may be responsible for obstructive heart defects ((rs2978663) OHDs) and hereditary anaemia (rs8190955) with different population frequencies. The TA haplotype was prominently found with a frequency of 0.533 in the studied population in comparison to haplotypes CG and CA.

## Discussion

Glutathione-disulfide reductase is a flavoprotein involved in the glutathione redox cycle maintaining proper function and preventing oxidative stress in RBCs. The literature reveals that deregulation of glutathione-disulfide reductase protein leads to activation of dormant cells and deregulation of the cell cycle. Changes in the GSR protein during the glutathione antioxidant defence system play a vital role in executing its function, but any nsSNPs in the *GSR* gene lead to aberrant conformations, which in turn leads to glutathione reductase deficiency. Therefore, it becomes necessary to identify the effects of deleterious nsSNPs of GSR and their association with various diseases.

This study aimed to determine the most deleterious nsSNPs and their effects on the function of GSR proteins, characterize the haplotype structure of these genes and investigate markers associated with disease. The 17 molecular consequences found in the ClinVar database, were further subjected to analysis of six insilico SNP prediction tools, SIFT, Mutation Tester, Polyphen-2, SNP&GO, PROVEAN, SNAP2, and PredictSNP to identify significant deleterious nsSNPs. Among the identified 17 input non-synonymous SNP data-set, 4 nsSNPs (V289A, R233C, A199Tand R153C) were found to be “damaging” with score ranging between 0.5–1.000 and 0.990–1.000 in protein structure and function by SIFT and PolyPhen 2 tools respectively and the remaining two nsSNPs were characterized as benign (Table 1). These result were further confirmed for their pathogenicity of *GSR* gene using SNPs&GO, Mutation tester, SNAP2, and PROVEAN server. The results obtained supported the results of SIFT and PolyPhen2 i.e. V289A, R233C, A199T, R153C are potential deleterious nsSNPs (Table 1).

These four nsSNPs revealed different domains of the protein, where two nsSNPs were positioned in the pyridine nucleotide-disulfide oxidoreductase domain that interacts with the GSR association domain^33^. One nsSNP was located in the FAD/NAD-binding domain, which serves as the FAD/NAD-binding domain involved in oxidative metabolism of a variety of hydrocarbons (rubredoxin reductase, putidaredoxin reductase, terpredoxin reductase, ferredoxin-NAD + reductase components of benzene 1,2-dioxygenase, toluene 1,2-dioxygenase, chlorobenzene dioxygenase, biphenyl dioxygenase), NADH oxidase and NADH peroxidase1, 2, 3. The fourth nsSNP was present in the mitochondrial apoptosis-inducing factor, the C-terminal domain, which is crucial for cell apoptosis^34^.

The stability of the protein structure is crucial for the proper function of the protein. Alternations in the stability of proteins may cause misfolding and degradation of proteins. Therefore, to study the structural and functional activity of proteins, protein stability studies were carried out by using the CUPSAT and DUET and I-Mutant 2.0 servers. The result revealed that the same mutations (V289A, R233C, A199T, R153C) were responsible for affecting the stability of the *GSR* protein structure (Table 2). Furthermore, evolutionary conservancy of the GSR protein sequence is vital to determine whether a mutation has any negative effect on the host. Using the ConSurf server, it was observed that highly deleterious nsSNPs with high conservation scores were located in highly conserved regions, therefore increasing the risk of hereditary anaemia by altering the GSR protein sequence.

The Project Hope server revealed that these four highly risky nsSNPs negatively affect the structure of the GSR protein, among which two nsSNPs were structural and two nsSNPs were functional residues according to ConSurf. The server revealed that the wild-type residues of R153C and R233C are more hydrophobic than the mutant residues, and these variations in size and hydrophobicity disrupt the H-bond interactions with the adjacent molecules due to loss of hydrophobic interactions in the core of the protein. Arginine (Wild-type) residue is strong basic positively charged amino acid in nature and has ability to form multiple hydrogen bonds and salt bridge whereas cysteine due to their high reactivity of S–H group can possible distort the regular structure of protein by interacting with other reactive groups. The strong charge of the wild residue places it towards the outer hydrophilic surfaces of the proteins^45,46^. Thus it can be seen as arginine plays a crucial role in stability and flexibility of protein, as this types of interaction can be elucidated by opposite charge attraction, length and flexibility of side chain, and the potential to produce excellent hydrogen-bonding geometries with other biomolecules like nucleobases and phosphate groups.

A to T substitutions are likely to be the outcome of single nucleotide polymorphisms (SNPs), the most prevalent class of genetic variation among individuals. The human genome contains at least 11 million SNPs, about 1% of which are non-synonymous coding SNPs (nsSNPs). Most supposedly deleterious nsSNPs affect protein stability rather than functionality, underlying the importance of structural consequences caused by residue substitutions. Considering the local nature of the cross- sheet model, single substitutions can substantially change the tendency of the modified amino acid sequence to form -sheet aggregates. Threonine residue is reported to induce aggregation in proteins and supports the beta-sheet structures by unique effects on interaction with its surroundings including the consequent 3D structure, stability and dynamic behavior, in contrast to alanine which supports formation of α- helices. Among the amino acids (Thr, Leu, Phe, Trp, Ile, Val, Tyr residues) in proteins having strong tendency to form intra- or intermolecular β-sheets, threonine is the only polar residue in the list of β -sheet inducers, and should thus exert unique effects on interaction with its surroundings. The consequent three-dimensional structure, stability and dynamic behavior of such protein regions are all tightly linked to the process of aggregation^47–51^. Similarly valine also support the formation of β-sheet. Also valine has been proven important for the oxidation of the reduced flavin by molecular oxygen in choline oxidase and it has been reported that replacement of Val464 with alanine in the enzyme results in a twofold decrease in the limiting rate constant for flavin reduction (k_red_) and less than fivefold decrease in the equilibrium constant for formation of the enzyme–substrate complex (K_d_)^52^. In the Val464Ala variant enzyme the substitution of the valine with an alanine has been reported to results in a 50-fold decrease in the bimolecular rate constant for reaction with oxygen, k_cat_/K_oxygen_. Further the authors have concluded that the presence of a nonpolar site is important for the oxidative half-reaction in which the enzyme-bound reduced flavin reacts with molecular oxygen to produce hydrogen peroxide and complete the catalytic cycle. It is proposed that the function of the nonpolar, aminoacyl side chain is to guide oxygen at the site where it subsequently will be activated to a superoxide species through electrostatic catalysis exerted by a positive charge^53^.

Linkage studies identified the 8p21 region as a susceptibility locus for obstructive heart defects (OHDs), hereditary anaemia. The LD pattern and haplotype structure for *GSR* in Han Chinese was characterized, and it revealed that there is LD across the *GSR* locus with little recombination. The rs3757918, rs8190955 and rs2978663 markers are loci enclosing a small part of the gene. Linkage disequilibrium has been reported between the common polymorphism found on *GSR* at positions 30619688 and 30627495. The analysis revealed that of the five nsSNPs identified, only three nsSNPs occurred and were linked in Han Chinese individuals. The results also indicated that only MAF (minor allele frequency) values of 0.467, 0.011 and 0.422 showed relatively strong linkage disequilibrium. The genotype of rs2978663 with the *GSR* gene increased the risk of occurrence of obstructive heart defects (OHDs). Right-sided and left-sided obstructive heart defects (OHDs) are subtypes of congenital heart defects in which the heart valves, arteries, or veins are abnormally narrow or blocked. Previous studies have suggested that the development of OHDs involves a complex interplay between genetic variants and maternal factors. Using data from 569 OHD case families and 1,644 control families enrolled in the National Birth Defects Prevention Study (NBDPS) between 1997 and 2008, we conducted an analysis to investigate the genetic effects of 877 single nucleotide polymorphisms (SNPs) in 60 candidate genes associated with the risk of OHDs and their interactions with maternal use of folic acid supplements and prepregnancy obesity. Applying log-linear models based on the hybrid design, we identified a SNP in the methylenetetrahydrofolate reductase (MTHFR) gene (C677T polymorphism) with a main genetic effect on the occurrence of OHDs. In addition, multiple SNPs in betaine-homocysteine methyltransferase (BHMT and BHMT2) were also identified to be associated with the occurrence of OHDs through significant main infant genetic effects and interaction effects with maternal use of folic acid supplements. We also identified multiple SNPs in glutamate-cysteine ligase, catalytic subunit (GCLC) and DNA (cytosine-5-)-methyltransferase 3 beta (DNMT3B) that were associated with an elevated risk of OHDs among obese women. Our findings suggested that the risk of OHDs was closely related to a combined effect of variations in genes in the folate, homocysteine, or glutathione/transsulfuration pathways, maternal use of folic acid supplements and prepregnancy. Obstructive heart defects associated with candidate genes, maternal obesity, and folic acid supplementation resolution in prepregnancy obese patients and maternal genotypes of SNPs in the *GSR* gene were associated with an increased risk of OHDs^35^. The genetic variant of *GSR* (rs8190955) was also found to be significantly associated with anaemia. This study demonstrates a potential connection between anaemia and oxidative stress, which could accelerate the production of ROS in addition to reducing the ability of the antioxidant defence system caused by SNPs of enzymes. LD and haplotype data should be useful in drug development and in understanding the genetic associations of *GSR* with adverse drug effects. These results provide an evidence of mutation leading to hereditary glutathione reductase deficiency and association with OHDs and can be further implemented for web lab studies.

## Materials and method

Hereditary red blood cell enzymopathies of *GSR* gene-related information were collected from the database Online Mendelian Inheritance In Man (OMIM)^13^ and other reported literature. The dataset of chromosome number and position of the *GSR* gene in the human genome was collected from the ClinVar database (https://www.ncbi.nlm.nih.gov/clinvar/) only for missense variants^25^. Missense variants were chosen for further analysis because of their higher impact on the structure and function of proteins.

### Identification of deleterious missense variants

Prediction of the deleterious effect of missense variants was performed by using seven different tools: Sorting Intolerant from Tolerant (SIFT)^26^ (http://sift.jcvi.org), Polymorphism Phenotypingv2 (PolyPhen-2)^27^ (http://genetics.bwh.harvard.edu/pph2/), SNP-GO^28^ (https://snps-and-go.biocomp.unibo.it/snps-and-go/), SNAP2^29^, Provean^30^, predictor of human deleterious single nucleotide Polymorphisms (PhD-SNP)^13,15^and MUTATION TASTER^31^ (http://www.mutationtaster.org/) tools. The SIFT tool is a sequence homology-based tool; if the score is equal to or less than 0.05, the nsSNPs are considered deleterious nsSNPs. The WHESS.db module of the PolyPhen-2 server is a sequence and structure evolutionary conservation based on classifying the damaging effect of amino acid substitution; if the score lies between 0.801–1.00, then the nsSNPs are considered probably damaging. The PROVEAN server provides a pairwise sequence alignment (PSA) score and identifies nonsynonymous variants. Single nucleotide polymorphisms & Gene Ontology (SNPs&GO) and predictors of human deleterious single nucleotide polymorphisms (PhD-SNPs) are both support vector machine (SVM)-based tools used to predict evolutionary information, protein sequences and functions if the given mutation can be classified as disease related. Furthermore, mutation tester servers were used to evaluate the DNA sequence variants for disease-causing potential. Mutation tester scores that ranged from < 0.5 were considered disease-causing.

### Identification of nsSNPs on the domains of GSR

The InterPro^32^ (https://www.ebi.ac.uk/interpro/) tool was used to identify the location of point mutations on the domains of glutathione-disulfide reductase protein, which can recognize motifs, active sites and domains of a protein.

### Predicting mutation effect on protein stability

Protein stability analysis of mutant proteins was performed using three different tools: CUPSAT, the DUET server and I-Mutant2^33^. CUPSAT was used to assess the effect of protein stability due to point mutation^34^. This tool uses structural environment-specific atom potential and torsion angle potentials to predict ΔΔG and the difference in the free energy between native and mutant proteins. Protein stability was also studied by using the DUET server^35^ and the I-Mutant 2.0 tool, which supports the vector machine SVM-based tool that predicts the change in protein stability upon single point mutation. The results obtained are in the form of stability of protein Gibbs free energy in the form of DDG values.

### Analysing of GSR protein evolutionary conservation

To understand the evolutionary conservation of the amino acids in the protein sequence, ConSurf^36^ (https://consurf.tau.ac.il) was used to analyses the phylogenetic relationships between homologous sequences. Considered those nsSNPs of GSR that were found to be conserved for further analyses.

### Prediction of the structural effect of nsSNPs on the human GSR protein

To identify the effect of the nsSNPs on the structure of the protein, HOPE (https://www3.cmbi.umcn.nl/hope) was used. HOPE is a web server that identifies the structural effects of point mutations in a protein sequence^37^. P00390 (UniProt-Accession Code of GSR) and the 4 SNPs were used individually as the input.

### LD and haplotype block analysis

LD plays a key role in mapping complex disease or trail-associated genes. Haplotype block provides information on patterns of genetic variation that are associated with health and disease, and it can be used to examine stretches of DNA near the SNP cluster to identify the gene or genes responsible for causing the disease. Linkage disequilibrium (LD) is used for the study of population genetics for the nonrandom association of alleles at different loci^38,39^. The Haploview tool^40^ from the MIT/Harvard Broad Institute was used to study the genotype data for quantitative genetic parameters such as LD, and haplotype block data of Han Chinese (CHB) were retrieved from the International Hapmap Project. The data were visualized and analysed for any linkages and generation of LD and haplotype blocks.

## Conclusion

GSR is a central enzyme in cellular antioxidant defence. The study of the functional SNPs of *GSR* provided significant insight into the deleterious effects of the nsSNPs identified in protein stability and cell functions. Two of the nsSNPs (rs2978663 and rs8190955) identified were also found to be associated with obstructive heart defects (OHDs) and hereditary anemia. It can be concluded that the LD and haplotype study unrevealed the relation of *GSR* with hereditary anaemia and OHD. Further that the understating of the oxidative stress pathways at molecular levels may be helpful in developing new interventions for the disease.

## Supplementary Information

Supplementary Information.

## Abbreviations

GSR: Glutathione-disulfide reductase
OHDs: Obstructive heart defects
nsSNPs: Non-synonymous single nucleotide polymorphisms
