# Supplementary material for: Identification, analysis of deleterious SNPs of the human GSR gene and their effects on the structure and functions of associated proteins and other diseases
Source: Sci Rep. 2022 Mar 31;12:5474. doi: 10.1038/s41598-022-09295-6 (PMC8971378; doi:10.1038/s41598-022-09295-6)
Supplement: Supplementary file 1 — Supplementary Information. [file 41598_2022_9295_MOESM1_ESM.docx]

**Identification, Analysis of deleterious SNPs of the human *GSR* gene and their effects on the structure and functions of associated proteins and other diseases**

Bharti Vyas^1^, Ratul Bhowmik^2^, Mymoona Akhter^2*^, Farhan Jalees Ahmad^1,2^

^1^School of Interdisciplinary Studies, Jamia Hamdard, New Delhi, India

^2^Department of Pharmaceutical Chemistry, School of Pharmaceutical Education and Research, Jamia Hamdard, New Delh, 110062, India

^*^Correspondence:

Mymoona Akhter

Professor

Department of Pharmaceutical Chemistry, School of Pharmaceutical Education and Research, Jamia Hamdard, New Delhi– 110062

E-mail: [makhtar@jamiahamdard.ac.in](mailto:makhtar@jamiahamdard.ac.in), **Orcid : 0000-0003-4380-3965**

**Author Email-Ids**

**Bharti Vyas:** [bhartivyas492@gmail.com](mailto:bhartivyas492@gmail.com)**, Orcid:** 0000-0002-9566-3004

Ratul Bhowmik: ss713724@gmail.com

Farhan Jalees Ahmad: fjahmad@jamiahamdard.ac.in,

**Table S1. Evolutionary conservancy of amino acids in GSR analyzed by Consurf**

| **POS** | **SEQ** | **3LATOM** | **SCORE** | **COLOR** | **CONFIDENCE INTERVAL** | **RESIDUE VARIETY** |  | **Amino Acid** |
| --- | --- | --- | --- | --- | --- | --- | --- | --- |
| 1 | A | - | 0.010 | 5* | -0.640, 0.384 | 7,4 | 5/150 | A,G |
| 2 | C | - | -0.387 | 6* | -1.111,-0.051 | 9,5 | 2/150 | C |
| 3 | R | - | -0.415 | 6* | -1.111,-0.051 | 9,5 | 2/150 | R |
| 4 | Q | - | -0.437 | 7* | -1.111,-0.051 | 9,5 | 2/150 | Q |
| 5 | E | - | -0.429 | 7* | -1.111,-0.051 | 9,5 | 2/150 | E |
| 6 | P | - | -0.394 | 6* | -1.111,-0.051 | 9,5 | 2/150 | P |
| 7 | Q | - | -0.437 | 7* | -1.111,-0.051 | 9,5 | 2/150 | Q |
| 8 | P | - | -0.394 | 6* | -1.111,-0.051 | 9,5 | 2/150 | P |
| 9 | Q | - | -0.437 | 7* | -1.111,-0.051 | 9,5 | 2/150 | Q |
| 10 | G | - | -0.387 | 6* | -1.111,-0.051 | 9,5 | 2/150 | G |
| 11 | P | - | -0.394 | 6* | -1.111,-0.051 | 9,5 | 2/150 | P |
| 12 | P | - | -0.825 | 8* | -1.210,-0.640 | 9,7 | 5/150 | P |
| 13 | P | - | -0.246 | 6* | -0.861, 0.073 | 8,5 | 5/150 | P,I |
| 14 | A | - | 0.756 | 2* | -0.161, 1.182 | 6,1 | 6/150 | S,G,A,K |
| 15 | A | - | 1.796 | 1 | 0.586, 3.122 | 3,1 | 12/150 | D,E,R,S,Q,A,P,L |
| 16 | G | - | 1.380 | 1 | 0.384, 1.711 | 4,1 | 16/150 | A,D,S,G,L,P |
| 17 | A | - | 1.224 | 1 | 0.384, 1.711 | 4,1 | 22/150 | R,G,S,D,H,C,E,K,A |
| 18 | V | VAL18:A | 1.802 | 1 | 0.838, 3.122 | 2,1 | 26/150 | E,H,S,I,M,G,R,A,N,T,V |
| 19 | A | ALA19:A | 0.916 | 2 | 0.384, 1.182 | 4,1 | 39/150 | F,K,E,Y,I,R,Q,A,V,L,T,N |
| 20 | S | SER20:A | -0.128 | 5 | -0.349,-0.051 | 6,5 | 132/150 | Q,A,X,V,T,D,H,F,C,E,K,R,G,S |
| 21 | Y | TYR21:A | 0.113 | 5 | -0.161, 0.216 | 6,4 | 144/150 | C,Y,A,F,V,T,L |
| 22 | D | ASP22:A | -1.260 | 9 | -1.302,-1.239 | 9,9 | 144/150 | E,D |
| 23 | Y | TYR23:A | -0.532 | 7 | -0.701,-0.431 | 7,7 | 144/150 | Y,F,I,M,L,V |
| 24 | L | LEU24:A | -0.426 | 6 | -0.576,-0.349 | 7,6 | 145/150 | Y,C,F,L,V,I |
| 25 | V | VAL25:A | -0.965 | 8 | -1.074,-0.908 | 9,8 | 145/150 | F,A,C,I,T,V |
| 26 | I | ILE26:A | -1.112 | 9 | -1.178,-1.074 | 9,9 | 145/150 | L,M,I |
| 27 | G | GLY27:A | -1.260 | 9 | -1.302,-1.239 | 9,9 | 145/150 | G |
| 28 | G | GLY28:A | -0.617 | 7 | -0.757,-0.506 | 8,7 | 145/150 | I,G,S,A |
| 29 | G | GLY29:A | -1.260 | 9 | -1.302,-1.239 | 9,9 | 145/150 | G |
| 30 | S | SER30:A | -1.254 | 9 | -1.291,-1.239 | 9,9 | 145/150 | P,T,S |
| 31 | G | GLY31:A | -1.084 | 9 | -1.178,-1.036 | 9,9 | 145/150 | G,A |
| 32 | G | GLY32:A | -1.260 | 9 | -1.302,-1.239 | 9,9 | 146/150 | G |
| 33 | L | LEU33:A | -0.674 | 7 | -0.811,-0.576 | 8,7 | 146/150 | A,I,M,V,T,L,S |
| 34 | A | ALA34:A | -1.034 | 9 | -1.111,-0.996 | 9,8 | 146/150 | G,R,S,A |
| 35 | S | SER35:A | -0.481 | 7 | -0.640,-0.431 | 7,7 | 146/150 | V,L,T,G,S,C,A |
| 36 | A | ALA36:A | -0.775 | 8 | -0.908,-0.701 | 8,7 | 146/150 | V,R,G,I,M,S,A |
| 37 | R | ARG37:A | -1.026 | 9 | -1.111,-0.996 | 9,8 | 146/150 | N,V,Q,S,I,R,E,K,F |
| 38 | R | ARG38:A | -0.430 | 7 | -0.576,-0.349 | 7,6 | 146/150 | V,L,T,Q,A,M,R,I,F,E,K |
| 39 | A | ALA39:A | -1.076 | 9 | -1.146,-1.036 | 9,9 | 146/150 | A,S,T,L |
| 40 | A | ALA40:A | -0.907 | 8 | -0.996,-0.861 | 8,8 | 146/150 | A,K,T,V,R,G,S |
| 41 | E | GLU41:A | 1.079 | 1 | 0.586, 1.182 | 3,1 | 146/150 | M,R,H,D,K,L,T,W,N,Q,A,I,G,S,E,V |
| 42 | L | LEU42:A | 0.989 | 2 | 0.586, 1.182 | 3,1 | 146/150 | A,N,W,L,T,K,E,Y,H,F,M,R,G |
| 43 | G | GLY43:A | -1.038 | 9 | -1.146,-0.996 | 9,8 | 146/150 | K,G,S,N |
| 44 | A | ALA44:A | -0.596 | 7 | -0.757,-0.506 | 8,7 | 146/150 | K,I,M,R,A,Q,V,L,T |
| 45 | R | ARG45:A | -0.224 | 6 | -0.431,-0.161 | 7,6 | 146/150 | T,R,S,N,H,K,A |
| 46 | A | ALA46:A | -1.004 | 9 | -1.111,-0.953 | 9,8 | 146/150 | S,M,T,V,A,C |
| 47 | A | ALA47:A | -0.484 | 7 | -0.640,-0.431 | 7,7 | 145/150 | G,M,I,L,V,A |
| 48 | V | VAL48:A | 0.199 | 4 | -0.051, 0.384 | 5,4 | 145/150 | A,C,I,V,L |
| 49 | V | VAL49:A | -0.353 | 6 | -0.506,-0.260 | 7,6 | 145/150 | A,V,T,L,F,C,M,I |
| 50 | E | GLU50:A | -1.210 | 9 | -1.266,-1.178 | 9,9 | 147/150 | R,E,D |
| 51 | S | SER51:A | 0.271 | 4 | -0.051, 0.384 | 5,4 | 147/150 | D,H,F,E,K,R,G,S,Q,A,P,T,V,N |
| 52 | H | HIS52:A | 1.035 | 1 | 0.586, 1.182 | 3,1 | 147/150 | R,M,D,F,H,Y,K,P,T,L,N,W,Q,A,G,S,E,V |
| 53 | K | LYS53:A | 0.317 | 4 | 0.073, 0.384 | 5,4 | 147/150 | R,G,E,Y,K,D,H,F,N,L,P,T,A |
| 54 | L | LEU54:A | 0.007 | 5 | -0.260, 0.073 | 6,5 | 147/150 | F,Y,M,I,S,Q,A,V,P,L,W |
| 55 | G | GLY55:A | -1.219 | 9 | -1.291,-1.178 | 9,9 | 147/150 | R,G |
| 56 | G | GLY56:A | -1.219 | 9 | -1.291,-1.178 | 9,9 | 148/150 | R,G |
| 57 | T | THR57:A | -1.246 | 9 | -1.291,-1.239 | 9,9 | 148/150 | R,T,C |
| 58 | C | CYS58:A | -1.260 | 9 | -1.302,-1.239 | 9,9 | 148/150 | C |
| 59 | V | VAL59:A | -1.221 | 9 | -1.266,-1.210 | 9,9 | 148/150 | A,L,V |
| 60 | N | ASN60:A | -1.116 | 9 | -1.178,-1.074 | 9,9 | 148/150 | K,H,N,I,L |
| 61 | V | VAL61:A | -0.842 | 8 | -0.953,-0.757 | 8,8 | 148/150 | R,M,I,K,V,L,N,Q,A |
| 62 | G | GLY62:A | -1.261 | 9 | -1.302,-1.239 | 9,9 | 148/150 | G |
| 63 | C | - | -1.260 | 9 | -1.302,-1.239 | 9,9 | 147/150 | X,C |
| 64 | V | VAL64:A | -1.095 | 9 | -1.178,-1.074 | 9,9 | 148/150 | I,V,T |
| 65 | P | PRO65:A | -1.268 | 9 | -1.302,-1.266 | 9,9 | 148/150 | P |
| 66 | K | LYS66:A | -1.282 | 9 | -1.302,-1.266 | 9,9 | 148/150 | K |
| 67 | K | LYS67:A | -1.282 | 9 | -1.302,-1.266 | 9,9 | 148/150 | K |
| 68 | V | VAL68:A | -0.669 | 7 | -0.811,-0.576 | 8,7 | 148/150 | H,F,A,M,I,V,L |
| 69 | M | MET69:A | -0.606 | 7 | -0.757,-0.506 | 8,7 | 148/150 | Y,C,F,S,I,M,T,L |
| 70 | W | TRP70:A | -0.636 | 7 | -0.811,-0.576 | 8,7 | 148/150 | H,F,Y,M,S,A,T,V,W |
| 71 | N | ASN71:A | -0.457 | 7 | -0.640,-0.349 | 7,6 | 148/150 | Q,L,N,W,H,F,C,Y,I,R,M,S |
| 72 | T | THR72:A | -0.666 | 7 | -0.811,-0.576 | 8,7 | 148/150 | A,L,V,T,C,F,S,G |
| 73 | A | ALA73:A | -0.783 | 8 | -0.908,-0.701 | 8,7 | 148/150 | S,G,A |
| 74 | V | VAL74:A | 0.320 | 4 | 0.073, 0.384 | 5,4 | 148/150 | N,V,L,T,A,Q,S,G,M,R,K,E,F,H,D |
| 75 | H | HIS75:A | 0.547 | 3 | 0.216, 0.838 | 4,2 | 148/150 | W,L,T,V,A,Q,I,M,Y,F,H |
| 76 | S | SER76:A | 0.153 | 4 | -0.161, 0.216 | 6,4 | 148/150 | N,V,P,A,Q,S,G,R,M,E,K,F,H |
| 77 | E | GLU77:A | 0.503 | 3 | 0.216, 0.586 | 4,3 | 148/150 | N,L,P,T,A,Q,S,M,G,R,K,E,H,D |
| 78 | F | PHE78:A | 0.938 | 2 | 0.586, 1.182 | 3,1 | 148/150 | G,I,S,E,V,R,M,F,H,D,K,Y,L,T,W,Q,A |
| 79 | M | MET79:A | 0.306 | 4 | -0.051, 0.384 | 5,4 | 148/150 | A,Q,N,L,V,E,F,M,I |
| 80 | H | HIS80:A | 0.887 | 2 | 0.586, 1.182 | 3,1 | 148/150 | R,I,S,H,D,K,E,T,P,L,N,Q,A |
| 81 | D | ASP81:A | 0.256 | 4 | -0.051, 0.384 | 5,4 | 148/150 | I,G,M,S,H,D,K,Y,E,T,L,V,N,Q,A |
| 82 | H | HIS82:A | -0.707 | 7 | -0.861,-0.640 | 8,7 | 148/150 | C,Y,A,H,S,G,M |
| 83 | A | ALA83:A | 2.384 | 1 | 1.182, 3.122 | 1,1 | 148/150 | S,R,I,M,G,K,E,H,D,L,T,P,V,A,Q |
| 84 | D | ASP84:A | 0.922 | 2 | 0.586, 1.182 | 3,1 | 148/150 | S,M,R,G,K,E,H,D,N,T,A,Q |
| 85 | Y | TYR85:A | -0.395 | 6 | -0.576,-0.260 | 7,6 | 148/150 | Y,H,F,V,L |
| 86 | G | GLY86:A | -1.124 | 9 | -1.210,-1.074 | 9,9 | 146/150 | G,P,A,K |
| 87 | F | PHE87:A | -0.740 | 8 | -0.908,-0.640 | 8,7 | 146/150 | F,H,Y,A,L,I,W |
| 88 | P | PRO88:A | 1.239 | 1 | 0.838, 1.711 | 2,1 | 146/150 | D,H,E,Y,K,G,R,S,Q,T,P,N |
| 89 | S | SER89:A | 1.414 | 1 | 0.838, 1.711 | 2,1 | 146/150 | V,S,I,G,E,N,T,P,L,A,Q,R,M,K,Y,F,H |
| 90 | C | CYS90:A | 3.118 | 1 | 1.711, 3.122 | 1,1 | 148/150 | R,K,Y,H,D,N,P,T,L,A,Q,S,G,E,C,V |
| 91 | E | GLU91:A | 3.088 | 1 | 1.711, 3.122 | 1,1 | 144/150 | A,Q,N,W,T,P,L,Y,K,D,H,R,V,C,E,S,I,G |
| 92 | G | GLY92:A | 1.932 | 1 | 1.182, 1.711 | 1,1 | 148/150 | V,S,I,G,C,E,W,N,P,T,L,A,Q,R,M,K,H,D |
| 93 | K | LYS93:A | 3.111 | 1 | 1.711, 3.122 | 1,1 | 147/150 | R,I,G,S,D,H,E,K,V,L,P,T,N,Q,A |
| 94 | F | PHE94:A | -0.463 | 7 | -0.640,-0.349 | 7,6 | 148/150 | I,S,F,H,Y,E,P,T,L,V |
| 95 | N | ASN95:A | -0.283 | 6 | -0.506,-0.161 | 7,6 | 148/150 | R,T,N,S,H,D,E |
| 96 | W | TRP96:A | -0.747 | 8 | -0.908,-0.640 | 8,7 | 148/150 | F,Y,V,W |
| 97 | R | ARG97:A | 2.983 | 1 | 1.711, 3.122 | 1,1 | 148/150 | D,H,E,K,R,G,S,Q,A,P,V,L,T,N |
| 98 | V | VAL98:A | 0.877 | 2 | 0.384, 1.182 | 4,1 | 148/150 | S,I,R,G,Y,K,D,H,L,T,V,A,Q |
| 99 | I | ILE99:A | -0.644 | 7 | -0.811,-0.576 | 8,7 | 148/150 | V,L,M,I,Y,A,F |
| 100 | K | LYS100:A | 0.014 | 5 | -0.260, 0.073 | 6,5 | 148/150 | L,V,T,A,Q,S,M,I,R,K,H |
| 101 | E | GLU101:A | 0.909 | 2 | 0.586, 1.182 | 3,1 | 148/150 | K,E,H,D,S,G,R,A,Q,N,P,V,L,T |
| 102 | K | LYS102:A | 0.066 | 5 | -0.161, 0.216 | 6,4 | 148/150 | A,Q,N,T,V,E,K,H,S,M,R,G |
| 103 | R | ARG103:A | -0.839 | 8 | -0.953,-0.757 | 8,8 | 148/150 | K,I,M,R,T,L,V,N |
| 104 | D | ASP104:A | 0.139 | 5 | -0.161, 0.216 | 6,4 | 148/150 | R,G,I,S,H,D,K,E,V,L,T,N,Q,A |
| 105 | A | ALA105:A | 0.551 | 3 | 0.216, 0.586 | 4,3 | 148/150 | S,G,M,I,R,K,D,H,N,V,T,A,Q |
| 106 | Y | TYR106:A | -0.932 | 8 | -1.036,-0.861 | 9,8 | 148/150 | R,H,F,D,Y,E,V,T,N,Q,A |
| 107 | V | VAL107:A | -0.688 | 7 | -0.811,-0.640 | 8,7 | 148/150 | V,L,I |
| 108 | S | SER108:A | 1.169 | 1 | 0.586, 1.182 | 3,1 | 148/150 | H,D,K,E,M,R,G,S,Q,A,T,V,L,N |
| 109 | R | ARG109:A | -0.954 | 8 | -1.074,-0.908 | 9,8 | 148/150 | N,R,G,M,S,H,F,D,K |
| 110 | L | LEU110:A | -0.820 | 8 | -0.953,-0.757 | 8,8 | 148/150 | L,V,I,S,N,F,A |
| 111 | N | ASN111:A | -0.799 | 8 | -0.908,-0.757 | 8,8 | 148/150 | R,V,T,N,S,H,E |
| 112 | A | ALA112:A | 1.382 | 1 | 0.838, 1.711 | 2,1 | 146/150 | Q,A,X,T,W,N,D,H,E,K,G,I,R,S |
| 113 | I | ILE113:A | -0.204 | 6 | -0.431,-0.051 | 7,5 | 148/150 | I,M,G,S,Y,K,V,L,N,W,A |
| 114 | Y | TYR114:A | -1.027 | 9 | -1.146,-0.953 | 9,8 | 148/150 | Y,H,F,W,T |
| 115 | Q | GLN115:A | 1.020 | 1 | 0.586, 1.182 | 3,1 | 147/150 | I,G,S,C,E,V,R,D,H,K,X,T,L,N,Q,A |
| 116 | N | ASN116:A | 1.036 | 1 | 0.586, 1.182 | 3,1 | 147/150 | A,Q,N,X,L,V,T,K,E,D,S,G,R |
| 117 | N | ASN117:A | 0.073 | 5 | -0.161, 0.216 | 6,4 | 148/150 | N,L,T,A,Q,M,R,Y,D,H,F,V,S,I,G,E |
| 118 | L | LEU118:A | -0.685 | 7 | -0.861,-0.576 | 8,7 | 148/150 | C,F,M,I,A,Q,W,V,L |
| 119 | T | THR119:A | 1.743 | 1 | 1.182, 1.711 | 1,1 | 148/150 | N,L,V,T,A,Q,S,R,M,G,K,E,H,D |
| 120 | K | LYS120:A | 0.399 | 4 | 0.073, 0.586 | 5,3 | 148/150 | L,T,N,Q,A,R,G,S,D,K,E |
| 121 | S | SER121:A | 0.018 | 5 | -0.260, 0.073 | 6,5 | 148/150 | N,P,V,T,L,A,Q,S,R,E,K,D,H,F |
| 122 | H | HIS122:A | 0.403 | 4 | 0.073, 0.586 | 5,3 | 148/150 | T,N,Q,A,R,G,S,D,H,E,C,K |
| 123 | I | ILE123:A | -0.660 | 7 | -0.811,-0.576 | 8,7 | 148/150 | V,T,I,C,A |
| 124 | E | GLU124:A | 0.886 | 2 | 0.384, 1.182 | 4,1 | 148/150 | I,M,R,S,D,H,E,K,V,T,Q,A |
| 125 | I | ILE125:A | 0.797 | 2 | 0.384, 0.838 | 4,2 | 147/150 | H,F,E,Y,R,I,M,S,A,T,X,L,V,W |
| 126 | I | ILE126:A | 0.599 | 3 | 0.216, 0.838 | 4,2 | 148/150 | M,I,Y,H,F,W,V,L,A |
| 127 | R | ARG127:A | 1.496 | 1 | 0.838, 1.711 | 2,1 | 148/150 | D,H,F,Y,K,R,Q,A,L,T,P,W,N,E,G,S,V |
| 128 | G | GLY128:A | 0.626 | 3 | 0.216, 0.838 | 4,2 | 147/150 | T,L,X,N,Q,A,I,M,G,S,D,F,H,C,E |
| 129 | H | HIS129:A | 0.807 | 2 | 0.384, 0.838 | 4,2 | 148/150 | H,F,K,Y,E,G,I,R,S,Q,A,P,L,T,W |
| 130 | A | ALA130:A | -0.798 | 8 | -0.908,-0.757 | 8,8 | 148/150 | G,A |
| 131 | A | ALA131:A | 1.443 | 1 | 0.838, 1.711 | 2,1 | 148/150 | M,I,R,S,H,E,K,V,T,N,Q,A |
| 132 | F | PHE132:A | 0.131 | 5 | -0.161, 0.216 | 6,4 | 148/150 | F,I,M,V,L |
| 133 | T | THR133:A | 0.952 | 2 | 0.586, 1.182 | 3,1 | 148/150 | H,D,K,Y,M,R,Q,A,L,T,N,E,C,I,S,V |
| 134 | S | SER134:A | 0.090 | 5 | -0.161, 0.216 | 6,4 | 148/150 | D,K,E,G,S,Q,A,T,N |
| 135 | D | ASP135:A | 1.598 | 1 | 0.838, 1.711 | 2,1 | 148/150 | S,G,K,E,H,D,N,T,V,P,A,Q |
| 136 | P | PRO136:A | 0.879 | 2* | -0.161, 1.711 | 6,1 | 5/150 | V,T,P |
| 137 | K | LYS137:A | -0.397 | 6* | -0.953,-0.051 | 8,5 | 5/150 | E,K |
| 138 | P | PRO138:A | -0.215 | 6 | -0.431,-0.161 | 7,6 | 148/150 | N,T,P,Q,S,G,R,K,E,H,D |
| 139 | T | THR139:A | -0.093 | 5 | -0.349, 0.073 | 6,5 | 148/150 | V,T,Q,A,M,I,R,S,D,F,H,C,E,K |
| 140 | I | ILE140:A | -0.249 | 6 | -0.431,-0.161 | 7,6 | 149/150 | I,M,L,V,A |
| 141 | E | GLU141:A | 1.093 | 1 | 0.586, 1.182 | 3,1 | 149/150 | T,L,V,N,Q,A,G,R,I,S,H,D,K,E,C |
| 142 | V | VAL142:A | -0.345 | 6 | -0.506,-0.260 | 7,6 | 149/150 | A,T,L,V,F,C,I,M,G |
| 143 | S | SER143:A | 1.307 | 1 | 0.838, 1.711 | 2,1 | 149/150 | T,N,Q,A,G,R,S,D,K,E |
| 144 | G | GLY144:A | 0.621 | 3 | 0.216, 0.838 | 4,2 | 149/150 | S,G,E,K,D,N,P,V,A |
| 145 | K | LYS145:A | 1.091 | 1 | 0.586, 1.182 | 3,1 | 149/150 | I,R,S,H,K,E,V,T,N,Q,A |
| 146 | K | LYS146:A | 2.924 | 1 | 1.711, 3.122 | 1,1 | 149/150 | L,P,V,T,Q,S,R,M,I,K,E,C,H,F |
| 147 | Y | TYR147:A | 1.291 | 1 | 0.838, 1.711 | 2,1 | 149/150 | K,Y,H,F,M,I,L,V |
| 148 | T | THR148:A | 0.073 | 5 | -0.161, 0.216 | 6,4 | 149/150 | A,Q,V,T,K,E,Y,F,S,R,G |
| 149 | A | ALA149:A | -0.986 | 8 | -1.074,-0.953 | 9,8 | 150/150 | G,T,S,A |
| 150 | P | PRO150:A | 0.859 | 2 | 0.384, 1.182 | 4,1 | 150/150 | Q,A,T,P,N,D,K,E,R,G,S |
| 151 | H | HIS151:A | 0.164 | 4 | -0.051, 0.216 | 5,4 | 150/150 | F,H,K,Y,I,R,S,T,N |
| 152 | I | ILE152:A | -1.001 | 9 | -1.074,-0.953 | 9,8 | 150/150 | L,T,V,M,I,F |
| 153 | L | LEU153:A | -0.591 | 7 | -0.757,-0.506 | 8,7 | 150/150 | L,T,V,M,I,C,A |
| 154 | I | ILE154:A | -0.548 | 7 | -0.701,-0.431 | 7,7 | 150/150 | I,V,L |
| 155 | A | ALA155:A | -1.245 | 9 | -1.291,-1.239 | 9,9 | 150/150 | S,A |
| 156 | T | THR156:A | -0.667 | 7 | -0.811,-0.576 | 8,7 | 150/150 | G,M,T,P,V,C |
| 157 | G | GLY157:A | -1.262 | 9 | -1.302,-1.239 | 9,9 | 150/150 | G |
| 158 | G | GLY158:A | -0.374 | 6 | -0.576,-0.260 | 7,6 | 150/150 | E,A,S,L,T,G,R |
| 159 | M | MET159:A | 0.957 | 2 | 0.586, 1.182 | 3,1 | 150/150 | Q,A,T,N,W,H,K,E,Y,I,G,M,R,S |
| 160 | P | PRO160:A | -0.896 | 8 | -1.036,-0.811 | 9,8 | 150/150 | A,E,S,I,V,P |
| 161 | S | SER161:A | 3.084 | 1 | 1.711, 3.122 | 1,1 | 150/150 | L,T,N,W,Q,A,M,R,F,H,Y,K,V,I,S,E |
| 162 | T | THR162:A | 1.552 | 1 | 0.838, 1.711 | 2,1 | 150/150 | S,M,I,R,K,Y,H,F,D,T,L,P,V,A,Q |
| 163 | P | PRO163:A | -0.559 | 7 | -0.757,-0.431 | 8,7 | 150/150 | A,T,P,V,L,E,K,F,S,M,G,I |
| 164 | H | HIS164:A | 1.503 | 1 | 0.586, 1.711 | 3,1 | 24/150 | E,K,D,H,F,S,Q,N,P,T |
| 165 | E | GLU165:A | 0.101 | 5* | -0.506, 0.586 | 7,3 | 8/150 | G,D,E |
| 166 | S | SER166:A | 0.152 | 4* | -0.506, 0.586 | 7,3 | 8/150 | S,T,E,K |
| 167 | Q | GLN167:A | 1.263 | 1 | 0.838, 1.711 | 2,1 | 150/150 | T,P,N,Q,A,I,G,M,R,S,H,D,K,E |
| 168 | I | ILE168:A | -0.046 | 5 | -0.260, 0.073 | 6,5 | 150/150 | A,L,T,V,F,Y,C,M,I |
| 169 | P | PRO169:A | -0.011 | 5 | -0.260, 0.073 | 6,5 | 150/150 | A,Q,N,V,P,E,K,D,H,S,R |
| 170 | G | GLY170:A | -1.134 | 9 | -1.239,-1.074 | 9,9 | 150/150 | G,N,D |
| 171 | A | ALA171:A | 0.831 | 2 | 0.384, 1.182 | 4,1 | 149/150 | H,F,Y,E,K,M,G,R,I,S,A,V,L,N |
| 172 | S | SER172:A | -0.531 | 7 | -0.701,-0.431 | 7,7 | 149/150 | L,N,Q,A,G,R,S,H,D,E |
| 173 | L | LEU173:A | 0.724 | 2 | 0.384, 0.838 | 4,2 | 149/150 | Y,E,D,H,F,L,V |
| 174 | G | GLY174:A | -0.067 | 5 | -0.260, 0.073 | 6,5 | 150/150 | G,I,T,V,F,A,C |
| 175 | I | ILE175:A | -0.643 | 7 | -0.757,-0.576 | 8,7 | 150/150 | A,V,L,T,W,F,C,E,R,M,I,G,S |
| 176 | T | THR176:A | -0.034 | 5 | -0.260, 0.073 | 6,5 | 150/150 | Y,H,D,S,G,I,N,T,V |
| 177 | S | SER177:A | -1.285 | 9 | -1.302,-1.291 | 9,9 | 150/150 | S |
| 178 | D | ASP178:A | -0.885 | 8 | -0.996,-0.811 | 8,8 | 150/150 | D,K,E,R,T,N |
| 179 | G | GLY179:A | -0.513 | 7 | -0.701,-0.431 | 7,7 | 150/150 | G,S,D,H,Q,E,A |
| 180 | F | PHE180:A | -0.170 | 6 | -0.349,-0.051 | 6,5 | 150/150 | I,M,V,L,A,C,F |
| 181 | F | PHE181:A | -1.181 | 9 | -1.266,-1.146 | 9,9 | 150/150 | F,L |
| 182 | Q | GLN182:A | 0.767 | 2 | 0.384, 0.838 | 4,2 | 150/150 | Q,A,T,V,L,W,N,D,F,H,E,Y,K,G,S |
| 183 | L | LEU183:A | -0.730 | 8 | -0.861,-0.640 | 8,7 | 150/150 | W,N,I,M,L,F,Q |
| 184 | E | GLU184:A | 0.867 | 2 | 0.384, 1.182 | 4,1 | 150/150 | D,K,E,G,R,S,Q,A,P,T,V,N |
| 185 | E | GLU185:A | 1.102 | 1 | 0.586, 1.182 | 3,1 | 150/150 | A,Q,N,T,V,Y,E,K,D,H,S,R |
| 186 | L | LEU186:A | 0.247 | 4 | -0.051, 0.384 | 5,4 | 150/150 | V,I,S,C,E,L,P,W,N,Q,A,R,M,D,F,Y,K |
| 187 | P | PRO187:A | -1.235 | 9 | -1.291,-1.210 | 9,9 | 150/150 | S,P |
| 188 | G | GLY188:A | 0.175 | 4 | -0.051, 0.384 | 5,4 | 150/150 | S,R,G,K,E,H,D,P,A,Q |
| 189 | R | ARG189:A | -0.144 | 6 | -0.349,-0.051 | 6,5 | 149/150 | Q,A,X,N,D,H,E,K,R,S |
| 190 | S | SER190:A | -0.129 | 5 | -0.349,-0.051 | 6,5 | 150/150 | I,M,S,F,C,T,L,V,W,A |
| 191 | V | VAL191:A | -0.111 | 5 | -0.349,-0.051 | 6,5 | 150/150 | G,I,T,V,L,A |
| 192 | I | ILE192:A | -0.440 | 7 | -0.640,-0.349 | 7,6 | 150/150 | L,T,V,I,M,C,F |
| 193 | V | VAL193:A | -0.357 | 6 | -0.506,-0.260 | 7,6 | 150/150 | N,W,V,L,A,Q,S,I,M,Y,E,F |
| 194 | G | GLY194:A | -1.262 | 9 | -1.302,-1.239 | 9,9 | 150/150 | G |
| 195 | A | ALA195:A | -0.349 | 6 | -0.576,-0.260 | 7,6 | 150/150 | A,S,G,T |
| 196 | G | GLY196:A | -1.146 | 9 | -1.239,-1.111 | 9,9 | 150/150 | G,S |
| 197 | Y | TYR197:A | -1.124 | 9 | -1.210,-1.074 | 9,9 | 150/150 | F,Y |
| 198 | I | ILE198:A | -1.195 | 9 | -1.266,-1.178 | 9,9 | 150/150 | I,V |
| 199 | A | ALA199:A | -0.772 | 8 | -0.908,-0.701 | 8,7 | 150/150 | A,G,S |
| 200 | V | VAL200:A | -0.414 | 6 | -0.576,-0.349 | 7,6 | 150/150 | M,I,S,C,L,V,T,N |
| 201 | E | GLU201:A | -1.283 | 9 | -1.302,-1.291 | 9,9 | 150/150 | E |
| 202 | M | MET202:A | -0.536 | 7 | -0.701,-0.431 | 7,7 | 150/150 | L,M,I,F,C |
| 203 | A | ALA203:A | -1.103 | 9 | -1.178,-1.074 | 9,9 | 150/150 | A,G,T,S |
| 204 | G | GLY204:A | -0.223 | 6 | -0.431,-0.051 | 7,5 | 150/150 | Q,A,T,N,H,C,G,S |
| 205 | I | ILE205:A | -0.715 | 8 | -0.861,-0.640 | 8,7 | 150/150 | F,A,L,V,I,M |
| 206 | L | LEU206:A | -0.338 | 6 | -0.506,-0.260 | 7,6 | 150/150 | L,V,M,Y,F |
| 207 | S | SER207:A | 0.189 | 4 | -0.051, 0.384 | 5,4 | 150/150 | K,C,H,S,R,G,A,Q,N,T |
| 208 | A | ALA208:A | -0.192 | 6 | -0.431,-0.051 | 7,5 | 150/150 | S,R,G,E,Y,K,N,T,A,Q |
| 209 | L | LEU209:A | -0.623 | 7 | -0.757,-0.506 | 8,7 | 150/150 | M,I,V,L,F,A,Y |
| 210 | G | GLY210:A | -1.089 | 9 | -1.178,-1.036 | 9,9 | 150/150 | M,G,N,S,K |
| 211 | S | SER211:A | 0.188 | 4 | -0.051, 0.384 | 5,4 | 150/150 | L,V,T,A,Q,S,I,G,C,Y |
| 212 | K | LYS212:A | 0.580 | 3 | 0.216, 0.838 | 4,2 | 150/150 | S,R,G,K,E,H,D,N,A,Q |
| 213 | T | THR213:A | -0.575 | 7 | -0.701,-0.506 | 7,7 | 150/150 | S,P,T,V,A |
| 214 | S | SER214:A | -0.123 | 5 | -0.349,-0.051 | 6,5 | 150/150 | H,F,D,K,E,I,R,M,S,Q,T,L,V,N |
| 215 | L | LEU215:A | -0.166 | 6 | -0.349,-0.051 | 6,5 | 150/150 | F,E,I,M,Q,V,T,L |
| 216 | M | MET216:A | 1.161 | 1 | 0.586, 1.182 | 3,1 | 150/150 | S,M,I,Y,C,F,N,T,V,L,A |
| 217 | I | ILE217:A | -0.264 | 6 | -0.431,-0.161 | 7,6 | 150/150 | V,G,I,S,E,C,L,T,N,Q,A,M,H,F,D,Y |
| 218 | R | ARG218:A | -1.165 | 9 | -1.239,-1.146 | 9,9 | 150/150 | F,H,L,T,M,R |
| 219 | H | HIS219:A | 0.602 | 3 | 0.216, 0.838 | 4,2 | 150/150 | Q,A,T,L,F,H,K,E,Y,R,G,S |
| 220 | D | ASP220:A | 0.880 | 2 | 0.384, 1.182 | 4,1 | 135/150 | K,E,H,D,S,G,R,A,Q,N,T,L,P |
| 221 | K | LYS221:A | 0.924 | 2 | 0.586, 1.182 | 3,1 | 149/150 | C,E,I,G,S,V,H,Y,K,R,M,Q,A,T,L,N,W |
| 222 | V | VAL222:A | -0.044 | 5 | -0.260, 0.073 | 6,5 | 150/150 | W,P,L,V,A,M,I,F |
| 223 | L | LEU223:A | -1.185 | 9 | -1.266,-1.146 | 9,9 | 150/150 | L,M |
| 224 | R | ARG224:A | -0.701 | 7 | -0.861,-0.640 | 8,7 | 150/150 | Q,A,P,T,N,W,D,K,E,G,R,S |
| 225 | S | SER225:A | 0.307 | 4 | -0.051, 0.384 | 5,4 | 150/150 | H,K,E,M,G,R,S,A,T,P,N |
| 226 | F | PHE226:A | -1.181 | 9 | -1.266,-1.146 | 9,9 | 150/150 | Q,H,F,Y |
| 227 | D | ASP227:A | -1.261 | 9 | -1.302,-1.239 | 9,9 | 150/150 | E,D |
| 228 | S | SER228:A | 1.079 | 1 | 0.586, 1.182 | 3,1 | 149/150 | A,Q,N,X,P,T,L,K,Y,H,D,R,M,V,E,S,G |
| 229 | M | MET229:A | 0.056 | 5 | -0.161, 0.216 | 6,4 | 150/150 | T,V,L,N,Q,A,G,M,I,S,D,F,Y,E |
| 230 | I | ILE230:A | 0.010 | 5 | -0.260, 0.073 | 6,5 | 150/150 | S,L,V,I,M,C,A |
| 231 | S | SER231:A | -0.168 | 6 | -0.349,-0.051 | 6,5 | 150/150 | I,G,R,S,H,Y,K,V,T,Q,A |
| 232 | T | THR232:A | 1.616 | 1 | 1.182, 1.711 | 1,1 | 150/150 | S,I,G,R,Y,E,K,D,H,N,V,L,T,A,Q |
| 233 | N | ASN233:A | 0.918 | 2 | 0.586, 1.182 | 3,1 | 150/150 | M,R,F,H,K,Y,T,L,N,Q,A,G,S,E,C,V |
| 234 | C | CYS234:A | 0.196 | 4 | -0.051, 0.384 | 5,4 | 150/150 | M,I,C,Y,T,V,L,A |
| 235 | T | THR235:A | 0.379 | 4 | 0.073, 0.586 | 5,3 | 150/150 | M,R,D,H,F,K,L,T,P,N,Q,A,I,G,S,E,V |
| 236 | E | GLU236:A | 0.731 | 2 | 0.384, 0.838 | 4,2 | 150/150 | F,D,K,E,G,R,S,Q,A,T,L,V,N |
| 237 | E | GLU237:A | 0.571 | 3 | 0.216, 0.838 | 4,2 | 150/150 | H,D,Y,R,M,Q,A,L,T,W,E,C,G,I,S,V |
| 238 | L | LEU238:A | -0.311 | 6 | -0.506,-0.260 | 7,6 | 149/150 | F,Y,M,I,S,A,X,L,V |
| 239 | E | GLU239:A | 1.723 | 1 | 1.182, 1.711 | 1,1 | 150/150 | V,G,I,S,E,C,T,L,N,Q,A,M,R,H,D,K |
| 240 | N | ASN240:A | 1.408 | 1 | 0.838, 1.711 | 2,1 | 150/150 | D,F,H,E,K,M,G,R,S,Q,A,V,L,T,N |
| 241 | A | ALA241:A | 0.684 | 3 | 0.384, 0.838 | 4,2 | 150/150 | A,Q,N,V,L,T,E,Y,K,D,H,S,R,I,G |
| 242 | G | GLY242:A | -1.173 | 9 | -1.266,-1.146 | 9,9 | 150/150 | P,G,H |
| 243 | V | VAL243:A | -0.292 | 6 | -0.506,-0.161 | 7,6 | 150/150 | I,M,C,F,P,T,L,V,A |
| 244 | E | GLU244:A | 1.489 | 1 | 0.838, 1.711 | 2,1 | 150/150 | T,P,V,N,Q,A,G,R,S,D,H,Y,E,K |
| 245 | V | VAL245:A | 0.389 | 4 | 0.073, 0.586 | 5,3 | 150/150 | Q,L,V,H,F,K,I,R,M |
| 246 | L | LEU246:A | 0.560 | 3 | 0.216, 0.838 | 4,2 | 150/150 | V,E,C,S,I,G,A,Q,N,W,L,T,K,H,M,R |
| 247 | K | LYS247:A | 0.608 | 3 | 0.216, 0.838 | 4,2 | 150/150 | G,S,E,C,V,R,F,H,Y,K,T,P,L,W,N,A |
| 248 | F | PHE248:A | 1.176 | 1 | 0.586, 1.182 | 3,1 | 150/150 | D,F,H,Y,C,E,K,R,G,S,Q,A,T,N |
| 249 | S | SER249:A | 1.080 | 1 | 0.586, 1.182 | 3,1 | 150/150 | V,E,C,S,Q,A,T,L,N,W,D,H,F,Y,K,R,M |
| 250 | Q | GLN250:A | 1.500 | 1 | 0.838, 1.711 | 2,1 | 150/150 | A,Q,N,T,L,P,K,H,D,M,R,V,E,S,G,I |
| 251 | V | VAL251:A | 0.414 | 4 | 0.073, 0.586 | 5,3 | 150/150 | L,P,V,A,M,I,H,F |
| 252 | K | LYS252:A | 2.454 | 1 | 1.182, 3.122 | 1,1 | 150/150 | F,D,K,E,M,R,I,G,S,Q,A,V,L,T,N |
| 253 | E | GLU253:A | 1.775 | 1 | 1.182, 1.711 | 1,1 | 150/150 | A,Q,N,W,L,T,E,K,D,S,G,M,R |
| 254 | V | VAL254:A | -0.391 | 6 | -0.576,-0.260 | 7,6 | 150/150 | F,A,M,I,L,V |
| 255 | K | LYS255:A | 0.543 | 3 | 0.216, 0.586 | 4,3 | 150/150 | R,G,I,S,D,F,H,E,K,V,T,N,Q,A |
| 256 | K | LYS256:A | 0.688 | 3 | 0.384, 0.838 | 4,2 | 150/150 | K,E,H,D,S,R,M,G,A,Q,N,P,T,L |
| 257 | T | THR257:A | 2.028 | 1 | 1.182, 1.711 | 1,1 | 145/150 | C,E,S,G,I,V,K,Y,H,D,R,A,Q,N,L,T,P |
| 258 | L | LEU258:A | 3.112 | 1 | 1.711, 3.122 | 1,1 | 149/150 | R,I,G,S,D,F,E,K,T,L,P,N,Q,A |
| 259 | S | SER259:A | 0.769 | 2 | 0.384, 0.838 | 4,2 | 149/150 | N,T,P,A,Q,S,G,R,I,E,K,D |
| 260 | G | GLY260:A | -0.797 | 8 | -0.996,-0.701 | 8,7 | 79/150 | D,E,G,N,S |
| 261 | L | LEU261:A | 0.630 | 3 | 0.216, 0.838 | 4,2 | 150/150 | F,K,Y,M,I,R,T,V,L,W |
| 262 | E | GLU262:A | 2.204 | 1 | 1.182, 3.122 | 1,1 | 150/150 | M,R,K,Y,H,D,W,N,L,T,A,Q,S,I,C,E,V |
| 263 | V | VAL263:A | 0.121 | 5 | -0.161, 0.216 | 6,4 | 150/150 | F,C,I,A,T,V,L,W,N |
| 264 | S | SER264:A | 1.815 | 1 | 1.182, 1.711 | 1,1 | 149/150 | V,E,C,G,I,S,Q,A,L,T,W,N,D,H,F,Y,K,R |
| 265 | M | MET265:A | 1.527 | 1 | 0.838, 1.711 | 2,1 | 150/150 | R,G,I,M,S,D,F,H,Y,C,T,L,V,W,A |
| 266 | V | VAL266:A | 1.491 | 1 | 0.838, 1.711 | 2,1 | 150/150 | L,V,P,T,N,Q,A,R,G,S,D,H,E,K |
| 267 | T | THR267:A | 0.426 | 4 | 0.073, 0.586 | 5,3 | 150/150 | Q,A,T,N,H,D,K,E,C,G,S |
| 268 | A | ALA268:A | 0.150 | 4 | -0.161, 0.384 | 6,4 | 150/150 | A,Q,N,T,K,E,C,H,D,S,R,G |
| 269 | V | VAL269:A | 1.437 | 1* | 0.216, 3.122 | 4,1 | 4/150 | V,F,D |
| 270 | P | PRO270:A | -0.590 | 7* | -1.146,-0.349 | 9,6 | 4/150 | P |
| 271 | G | GLY271:A | 1.160 | 1* | 0.073, 1.711 | 5,1 | 4/150 | G,N,E |
| 272 | R | ARG272:A | 0.721 | 2* | -0.260, 1.182 | 6,1 | 4/150 | Q,K,R |
| 273 | L | LEU273:A | 1.115 | 1* | -0.051, 1.711 | 5,1 | 4/150 | K,E,L |
| 274 | P | PRO274:A | 0.279 | 4* | -0.640, 0.838 | 7,2 | 4/150 | E,P |
| 275 | V | VAL275:A | 2.759 | 1 | 1.711, 3.122 | 1,1 | 23/150 | D,K,G,Q,A,P,T,V,N |
| 276 | M | MET276:A | 1.770 | 1 | 0.838, 3.122 | 2,1 | 27/150 | V,T,A,Q,G,M,I,R,E,K |
| 277 | T | THR277:A | 1.455 | 1 | 0.586, 1.711 | 3,1 | 37/150 | K,C,E,H,S,R,A,Q,N,V,T,L |
| 278 | M | MET278:A | 1.902 | 1 | 1.182, 1.711 | 1,1 | 150/150 | N,L,P,T,A,Q,R,M,Y,K,D,H,F,V,S,G,I,C,E |
| 279 | I | ILE279:A | 2.106 | 1 | 1.182, 3.122 | 1,1 | 150/150 | V,S,G,I,E,C,N,L,P,T,A,Q,R,M,K,D,H,F |
| 280 | P | PRO280:A | 2.816 | 1 | 1.711, 3.122 | 1,1 | 150/150 | E,G,I,S,V,F,H,D,K,Y,M,R,Q,A,P,L,T,N |
| 281 | D | ASP281:A | 1.500 | 1 | 0.838, 1.711 | 2,1 | 150/150 | H,D,K,R,M,Q,A,T,P,L,N,E,I,G,S,V |
| 282 | V | VAL282:A | 0.748 | 2 | 0.384, 0.838 | 4,2 | 150/150 | S,G,I,C,Y,F,N,T,V,A |
| 283 | D | ASP283:A | -0.634 | 7 | -0.757,-0.576 | 8,7 | 150/150 | G,T,N,S,Q,D,E |
| 284 | C | CYS284:A | 1.413 | 1 | 0.838, 1.711 | 2,1 | 150/150 | T,V,L,A,Q,S,M,G,I,R,K,C,E,H |
| 285 | L | LEU285:A | -0.580 | 7 | -0.757,-0.506 | 8,7 | 150/150 | I,V,L,F,A |
| 286 | L | LEU286:A | -0.127 | 5 | -0.349,-0.051 | 6,5 | 150/150 | F,I,M,V,L |
| 287 | W | TRP287:A | 0.681 | 3 | 0.216, 0.838 | 4,2 | 150/150 | A,W,L,T,V,C,Y,F,S,G,M,I |
| 288 | A | ALA288:A | -1.183 | 9 | -1.239,-1.146 | 9,9 | 150/150 | S,G,C,A |
| 289 | I | ILE289:A | -0.659 | 7 | -0.811,-0.576 | 8,7 | 150/150 | L,T,V,I,A |
| 290 | G | GLY290:A | -1.262 | 9 | -1.302,-1.239 | 9,9 | 150/150 | G |
| 291 | R | ARG291:A | -1.280 | 9 | -1.302,-1.266 | 9,9 | 150/150 | R |
| 292 | V | VAL292:A | 2.844 | 1 | 1.711, 3.122 | 1,1 | 150/150 | A,Q,N,L,T,V,E,K,D,H,S,M,I,G,R |
| 293 | P | PRO293:A | -0.540 | 7 | -0.701,-0.431 | 7,7 | 150/150 | S,P,T,G,A |
| 294 | N | ASN294:A | -0.325 | 6 | -0.506,-0.260 | 7,6 | 150/150 | D,H,C,Y,E,K,R,M,S,Q,A,L,T,N,W |
| 295 | T | THR295:A | -0.076 | 5 | -0.260, 0.073 | 6,5 | 150/150 | I,M,S,F,H,V,L,T,N,A |
| 296 | K | LYS296:A | 1.624 | 1 | 1.182, 1.711 | 1,1 | 148/150 | Q,A,T,V,N,D,H,E,K,R,G,S |
| 297 | D | ASP297:A | 1.200 | 1 | 0.586, 1.182 | 3,1 | 150/150 | R,G,S,H,D,K,E,T,P,V,N,Q,A |
| 298 | L | LEU298:A | -0.757 | 8 | -0.908,-0.701 | 8,7 | 150/150 | Y,L,M,I |
| 299 | S | SER299:A | 0.161 | 4 | -0.161, 0.384 | 6,4 | 149/150 | K,E,H,F,D,S,R,G,A,Q,N |
| 300 | L | LEU300:A | -1.028 | 9 | -1.146,-0.953 | 9,8 | 150/150 | L,I,C,A,F |
| 301 | N | ASN301:A | -0.045 | 5 | -0.260, 0.073 | 6,5 | 150/150 | A,Q,N,P,V,E,K,D,S,G |
| 302 | K | LYS302:A | 1.487 | 1 | 0.838, 1.711 | 2,1 | 150/150 | A,Q,N,T,V,Y,C,E,K,D,H,S,G,R,I |
| 303 | L | LEU303:A | 0.445 | 3 | 0.073, 0.586 | 5,3 | 148/150 | F,C,I,A,V,P,T,L |
| 304 | G | GLY304:A | -0.214 | 6 | -0.431,-0.051 | 7,5 | 150/150 | Q,A,N,D,K,C,E,G,S |
| 305 | I | ILE305:A | -0.799 | 8 | -0.908,-0.757 | 8,8 | 150/150 | I,P,V,L |
| 306 | Q | GLN306:A | 1.165 | 1 | 0.586, 1.182 | 3,1 | 150/150 | S,R,G,I,K,E,H,D,N,P,L,T,V,A,Q |
| 307 | T | THR307:A | 0.900 | 2 | 0.384, 1.182 | 4,1 | 150/150 | S,I,R,M,Y,C,F,H,P,L,V,T,A,Q |
| 308 | D | ASP308:A | 0.865 | 2 | 0.384, 1.182 | 4,1 | 146/150 | D,H,E,K,R,M,G,S,A,L,T,N |
| 309 | D | ASP309:A | 2.142 | 1 | 1.182, 3.122 | 1,1 | 150/150 | K,E,F,H,D,S,R,I,G,A,Q,W,N,T,P |
| 310 | K | LYS310:A | 1.395 | 1 | 0.838, 1.711 | 2,1 | 150/150 | V,G,S,E,T,L,N,Q,A,M,R,D,F,H,Y,K |
| 311 | G | GLY311:A | -0.582 | 7 | -0.757,-0.506 | 8,7 | 148/150 | N,Q,A,R,G,S,D,E,K |
| 312 | H | HIS312:A | 0.547 | 3 | 0.216, 0.586 | 4,3 | 150/150 | T,V,L,N,Q,A,G,R,M,S,F,H,E,Y,K |
| 313 | I | ILE313:A | -0.461 | 7 | -0.640,-0.349 | 7,6 | 150/150 | I,L,V,K |
| 314 | I | ILE314:A | 3.117 | 1 | 1.711, 3.122 | 1,1 | 150/150 | Q,A,L,P,T,V,H,D,K,Y,C,E,R,I,S |
| 315 | V | VAL315:A | -0.893 | 8 | -0.996,-0.811 | 8,8 | 150/150 | A,T,V,I |
| 316 | D | ASP316:A | -0.882 | 8 | -0.996,-0.811 | 8,8 | 150/150 | N,G,T,E,D |
| 317 | E | GLU317:A | 0.674 | 3 | 0.384, 0.838 | 4,2 | 150/150 | Q,A,L,P,N,D,H,C,E,K,R,G,S |
| 318 | F | PHE318:A | 1.676 | 1 | 1.182, 1.711 | 1,1 | 150/150 | T,L,W,N,Q,A,R,M,D,H,F,Y,K,V,G,S,E,C |
| 319 | Q | GLN319:A | -0.626 | 7 | -0.757,-0.576 | 8,7 | 150/150 | E,Y,C,H,F,D,S,M,A,Q,N,L |
| 320 | N | ASN320:A | 0.077 | 5 | -0.161, 0.216 | 6,4 | 150/150 | K,Y,C,E,R,G,S,Q,A,T,V,N |
| 321 | T | THR321:A | -0.982 | 8 | -1.074,-0.953 | 9,8 | 150/150 | A,S,T |
| 322 | N | ASN322:A | -0.102 | 5 | -0.349,-0.051 | 6,5 | 150/150 | D,K,R,G,S,Q,A,P,V,T,N |
| 323 | V | VAL323:A | -0.250 | 6 | -0.431,-0.161 | 7,6 | 150/150 | A,Q,N,V,L,T,E,C,H,F,D,R,I |
| 324 | K | LYS324:A | 1.373 | 1 | 0.838, 1.711 | 2,1 | 150/150 | P,N,Q,A,G,R,S,H,D,K,E |
| 325 | G | GLY325:A | -0.259 | 6 | -0.431,-0.161 | 7,6 | 150/150 | D,H,Y,G,R,S,A,W,N |
| 326 | I | ILE326:A | -0.863 | 8 | -0.953,-0.811 | 8,8 | 150/150 | V,L,I |
| 327 | Y | TYR327:A | -0.127 | 5 | -0.349,-0.051 | 6,5 | 150/150 | L,S,W,H,F,Y |
| 328 | A | ALA328:A | -1.124 | 9 | -1.210,-1.074 | 9,9 | 150/150 | C,A,S |
| 329 | V | VAL329:A | -0.226 | 6 | -0.431,-0.161 | 7,6 | 150/150 | L,V,I,A,F |
| 330 | G | GLY330:A | -1.262 | 9 | -1.302,-1.239 | 9,9 | 150/150 | G |
| 331 | D | ASP331:A | -1.280 | 9 | -1.302,-1.266 | 9,9 | 150/150 | D |
| 332 | V | VAL332:A | -0.658 | 7 | -0.811,-0.576 | 8,7 | 150/150 | A,C,I,M,V,L,N |
| 333 | C | CYS333:A | -0.290 | 6 | -0.506,-0.161 | 7,6 | 150/150 | I,G,S,E,Y,C,V,L,T,N,Q,A |
| 334 | G | GLY334:A | 0.145 | 4 | -0.161, 0.216 | 6,4 | 150/150 | A,N,T,K,E,H,D,S,G |
| 335 | K | LYS335:A | -0.073 | 5 | -0.260, 0.073 | 6,5 | 150/150 | E,Y,K,D,H,I,M,R,G,A,Q,N,T,V,L |
| 336 | A | ALA336:A | 0.659 | 3 | 0.216, 0.838 | 4,2 | 150/150 | Q,A,P,T,L,V,D,F,Y,K,I,R,M |
| 337 | L | LEU337:A | -0.532 | 7 | -0.701,-0.431 | 7,7 | 150/150 | N,L,P,A,Q,M,E,H |
| 338 | L | LEU338:A | -1.273 | 9 | -1.302,-1.266 | 9,9 | 150/150 | L |
| 339 | T | THR339:A | -1.282 | 9 | -1.302,-1.266 | 9,9 | 150/150 | T |
| 340 | P | PRO340:A | -1.268 | 9 | -1.302,-1.266 | 9,9 | 150/150 | P |
| 341 | V | VAL341:A | -1.094 | 9 | -1.178,-1.036 | 9,9 | 149/150 | M,I,A,W,V,P,T,L |
| 342 | A | ALA342:A | -1.282 | 9 | -1.302,-1.266 | 9,9 | 149/150 | A |
| 343 | I | ILE343:A | -0.976 | 8 | -1.074,-0.908 | 9,8 | 149/150 | I,L,T,V,K |
| 344 | A | ALA344:A | -0.580 | 7 | -0.701,-0.506 | 7,7 | 150/150 | M,R,G,S,H,D,K,E,T,L,N,Q,A |
| 345 | A | ALA345:A | -1.076 | 9 | -1.146,-1.036 | 9,9 | 150/150 | E,A,D,Q,S,V |
| 346 | G | GLY346:A | -0.845 | 8 | -0.996,-0.757 | 8,8 | 150/150 | A,G |
| 347 | R | ARG347:A | -0.596 | 7 | -0.757,-0.506 | 8,7 | 150/150 | Q,A,V,L,T,W,H,K,R,G,M,S |
| 348 | K | LYS348:A | -0.234 | 6 | -0.431,-0.161 | 7,6 | 150/150 | S,R,K,Y,C,N,W,V,T,L,A,Q |
| 349 | L | LEU349:A | -0.443 | 7 | -0.640,-0.349 | 7,6 | 150/150 | L,V,I,M,F |
| 350 | A | ALA350:A | -0.649 | 7 | -0.811,-0.576 | 8,7 | 150/150 | H,M,I,G,S,A,L,V |
| 351 | H | HIS351:A | -0.161 | 6 | -0.349,-0.051 | 6,5 | 150/150 | K,E,H,D,S,R,A,Q,N |
| 352 | R | ARG352:A | -0.718 | 8 | -0.861,-0.640 | 8,7 | 150/150 | Y,H,S,R,G,Q,N,T,L |
| 353 | L | LEU353:A | -0.277 | 6 | -0.506,-0.161 | 7,6 | 150/150 | R,I,S,F,H,E,V,T,L,W,Q,A |
| 354 | F | PHE354:A | -0.292 | 6 | -0.506,-0.161 | 7,6 | 150/150 | F,C,Y,I,Q,A,V,L |
| 355 | E | GLU355:A | 0.501 | 3 | 0.216, 0.586 | 4,3 | 150/150 | Q,A,T,L,N,D,K,C,E,G,R,S |
| 356 | Y | TYR356:A | 1.163 | 1 | 0.586, 1.182 | 3,1 | 150/150 | L,T,P,N,Q,A,G,R,S,H,D,K,E,Y |
| 357 | K | LYS357:A | 1.060 | 1 | 0.586, 1.182 | 3,1 | 150/150 | Y,K,D,H,R,M,A,Q,N,T,P,E,S,I,G,V |
| 358 | E | GLU358:A | 1.064 | 1 | 0.586, 1.182 | 3,1 | 149/150 | F,D,K,E,R,G,I,S,Q,A,T,V,L,P,N |
| 359 | D | ASP359:A | 1.417 | 1 | 0.838, 1.711 | 2,1 | 59/150 | K,Y,E,D,S,G,I,A,Q,N,T,P |
| 360 | S | SER360:A | 0.743 | 2 | 0.384, 0.838 | 4,2 | 150/150 | V,S,I,G,C,E,W,P,L,T,A,Q,R,M,K,Y,H,D |
| 361 | K | LYS361:A | 1.565 | 1 | 0.838, 1.711 | 2,1 | 149/150 | W,L,P,T,X,A,Q,M,R,Y,K,D,H,V,S,I,C,E |
| 362 | L | LEU362:A | 0.206 | 4 | -0.051, 0.384 | 5,4 | 150/150 | I,M,C,F,W,T,P,V,L,A |
| 363 | D | ASP363:A | -0.569 | 7 | -0.701,-0.506 | 7,7 | 150/150 | S,G,R,C,E,H,D,N,V |
| 364 | Y | TYR364:A | -0.563 | 7 | -0.701,-0.431 | 7,7 | 149/150 | Q,A,L,X,P,H,F,Y |
| 365 | N | ASN365:A | 1.685 | 1 | 1.182, 1.711 | 1,1 | 150/150 | T,L,W,N,Q,A,M,I,R,G,S,H,D,K,E |
| 366 | N | ASN366:A | 0.387 | 4 | 0.073, 0.586 | 5,3 | 150/150 | V,E,C,I,G,S,Q,A,T,L,N,F,H,D,K,Y,M |
| 367 | I | ILE367:A | -0.707 | 7 | -0.861,-0.640 | 8,7 | 150/150 | A,I,T,V |
| 368 | P | PRO368:A | -0.166 | 6 | -0.431,-0.051 | 7,5 | 150/150 | A,I,P,T,S |
| 369 | T | THR369:A | -0.781 | 8 | -0.908,-0.701 | 8,7 | 150/150 | K,A,C,S,T |
| 370 | V | VAL370:A | -1.163 | 9 | -1.239,-1.146 | 9,9 | 150/150 | V,T,C,A |
| 371 | V | VAL371:A | -0.937 | 8 | -1.036,-0.861 | 9,8 | 150/150 | T,V,I |
| 372 | F | PHE372:A | -1.244 | 9 | -1.291,-1.210 | 9,9 | 149/150 | C,F |
| 373 | S | SER373:A | -0.767 | 8 | -0.908,-0.701 | 8,7 | 150/150 | F,C,A,T,M,G,S |
| 374 | H | HIS374:A | -0.519 | 7 | -0.701,-0.431 | 7,7 | 150/150 | R,I,M,S,D,H,E,T,P,L,N,Q,A |
| 375 | P | PRO375:A | -1.199 | 9 | -1.266,-1.178 | 9,9 | 150/150 | L,P,Q |
| 376 | P | PRO376:A | -0.666 | 7 | -0.811,-0.576 | 8,7 | 150/150 | E,S,Q,A,P,V,T,N |
| 377 | I | ILE377:A | -0.271 | 6 | -0.431,-0.161 | 7,6 | 150/150 | F,Y,C,I,G,M,A,T,V,L |
| 378 | G | GLY378:A | -0.529 | 7 | -0.701,-0.431 | 7,7 | 150/150 | G,S,A |
| 379 | T | THR379:A | -0.548 | 7 | -0.701,-0.431 | 7,7 | 150/150 | A,Q,N,T,V,C,S,G,M,R |
| 380 | V | VAL380:A | -0.645 | 7 | -0.811,-0.576 | 8,7 | 150/150 | V,T,L,I,M,C |
| 381 | G | GLY381:A | -1.262 | 9 | -1.302,-1.239 | 9,9 | 150/150 | G |
| 382 | L | LEU382:A | -0.428 | 6 | -0.640,-0.349 | 7,6 | 150/150 | W,V,L,M,I,R,Y,F |
| 383 | T | THR383:A | -0.584 | 7 | -0.701,-0.506 | 7,7 | 150/150 | N,S,G,T,A |
| 384 | E | GLU384:A | -1.250 | 9 | -1.291,-1.239 | 9,9 | 150/150 | T,Q,E |
| 385 | D | ASP385:A | 0.507 | 3 | 0.216, 0.586 | 4,3 | 150/150 | N,P,T,V,A,Q,S,I,G,E,K,D,H |
| 386 | E | GLU386:A | 0.113 | 5 | -0.161, 0.216 | 6,4 | 150/150 | R,I,S,D,K,E,Q,A |
| 387 | A | ALA387:A | -1.282 | 9 | -1.302,-1.266 | 9,9 | 150/150 | A |
| 388 | I | ILE388:A | 0.438 | 3 | 0.073, 0.586 | 5,3 | 149/150 | I,M,G,R,K,C,E,H,N,V,T,L,A,Q |
| 389 | H | HIS389:A | 1.079 | 1 | 0.586, 1.182 | 3,1 | 150/150 | A,Q,N,V,L,T,E,K,D,H,S,G,R,I |
| 390 | K | LYS390:A | 1.284 | 1 | 0.838, 1.711 | 2,1 | 150/150 | N,L,V,T,A,Q,S,M,R,Y,E,K,D,H |
| 391 | Y | TYR391:A | 0.944 | 2 | 0.586, 1.182 | 3,1 | 134/150 | Y,C,H,F,S,I,R,G,A,V,T,L |
| 392 | G | GLY392:A | -0.603 | 7 | -0.811,-0.431 | 8,7 | 72/150 | G,P,A,K,Q,D |
| 393 | I | ILE393:A | 0.900 | 2 | 0.384, 1.182 | 4,1 | 57/150 | D,H,E,K,G,I,R,S,Q,A,N |
| 394 | E | GLU394:A | 1.162 | 1 | 0.586, 1.182 | 3,1 | 150/150 | Q,A,T,P,N,D,H,E,C,K,R,I,G,S |
| 395 | N | ASN395:A | 1.315 | 1 | 0.838, 1.711 | 2,1 | 150/150 | K,E,C,H,D,S,G,R,A,Q,N,V,P,T |
| 396 | V | VAL396:A | 0.164 | 4 | -0.051, 0.216 | 5,4 | 149/150 | Y,A,X,L,V,I |
| 397 | K | LYS397:A | 0.056 | 5 | -0.161, 0.216 | 6,4 | 150/150 | L,V,T,Q,A,R,S,H,D,K,Y,E |
| 398 | T | THR398:A | -0.190 | 6 | -0.431,-0.051 | 7,5 | 150/150 | A,T,L,V,Y,C,I,S |
| 399 | Y | TYR399:A | -0.631 | 7 | -0.811,-0.506 | 8,7 | 150/150 | S,R,Y,E,K,F,H |
| 400 | S | SER400:A | 0.952 | 2 | 0.586, 1.182 | 3,1 | 150/150 | K,C,E,Y,H,S,R,I,A,Q,N,L,V,T |
| 401 | T | THR401:A | -0.496 | 7 | -0.640,-0.431 | 7,7 | 150/150 | S,N,T,G,R,A,K |
| 402 | S | SER402:A | 0.949 | 2 | 0.586, 1.182 | 3,1 | 150/150 | E,S,I,G,V,K,Y,H,F,D,R,A,Q,N,L,T |
| 403 | F | PHE403:A | -1.179 | 9 | -1.239,-1.146 | 9,9 | 150/150 | Y,F,T |
| 404 | T | THR404:A | -0.174 | 6 | -0.349,-0.051 | 6,5 | 150/150 | N,W,T,L,V,A,Q,S,G,I,R,K,F |
| 405 | P | PRO405:A | -0.089 | 5 | -0.349, 0.073 | 6,5 | 150/150 | A,D,N,S,G,T,P |
| 406 | M | MET406:A | -0.837 | 8 | -0.953,-0.757 | 8,8 | 150/150 | F,M,I,T,L |
| 407 | Y | TYR407:A | -0.262 | 6 | -0.431,-0.161 | 7,6 | 150/150 | L,V,Q,A,M,G,R,S,H,F,Y,E,K |
| 408 | H | HIS408:A | 0.493 | 3 | 0.216, 0.586 | 4,3 | 150/150 | N,W,T,L,A,Q,S,G,R,E,C,Y,H,F,D |
| 409 | A | ALA409:A | -0.442 | 7 | -0.576,-0.349 | 7,6 | 150/150 | R,I,G,S,F,D,L,V,T,Q,A |
| 410 | V | VAL410:A | 0.035 | 5 | -0.260, 0.216 | 6,4 | 150/150 | L,V,M,I,F,Q,A |
| 411 | T | THR411:A | 0.021 | 5 | -0.161, 0.073 | 6,5 | 150/150 | I,M,G,S,F,E,P,L,V,T,N,A |
| 412 | K | LYS412:A | 1.123 | 1 | 0.586, 1.182 | 3,1 | 150/150 | N,L,P,T,A,Q,S,G,R,I,K,E,C,H,D |
| 413 | R | ARG413:A | 0.600 | 3 | 0.216, 0.838 | 4,2 | 150/150 | C,E,S,I,G,V,Y,K,D,H,M,R,A,Q,N,L,T |
| 414 | K | LYS414:A | 0.979 | 2 | 0.586, 1.182 | 3,1 | 150/150 | G,R,S,H,D,K,E,V,T,P,N,Q,A |
| 415 | T | THR415:A | -0.294 | 6 | -0.506,-0.161 | 7,6 | 150/150 | Q,A,L,T,V,P,N,D,H,E,M,R,G,I,S |
| 416 | K | LYS416:A | 0.143 | 4 | -0.161, 0.216 | 6,4 | 150/150 | H,F,K,Y,M,R,Q,A,P,T,L |
| 417 | C | CYS417:A | -0.159 | 6 | -0.349,-0.051 | 6,5 | 150/150 | V,T,N,A,I,G,M,S,C |
| 418 | V | VAL418:A | 0.794 | 2 | 0.384, 0.838 | 4,2 | 150/150 | A,V,T,L,D,F,H,Y,E,K,M,R,G,I,S |
| 419 | M | MET419:A | -0.500 | 7 | -0.640,-0.431 | 7,7 | 150/150 | Y,H,F,I,M,A,T,V,L |
| 420 | K | LYS420:A | -1.282 | 9 | -1.302,-1.266 | 9,9 | 150/150 | K |
| 421 | M | MET421:A | -0.605 | 7 | -0.757,-0.506 | 8,7 | 150/150 | A,I,M,V,L |
| 422 | V | VAL422:A | -0.206 | 6 | -0.431,-0.051 | 7,5 | 150/150 | V,L,I |
| 423 | C | CYS423:A | -0.746 | 8 | -0.861,-0.640 | 8,7 | 149/150 | A,C,F,V,L,T |
| 424 | A | ALA424:A | 0.977 | 2 | 0.586, 1.182 | 3,1 | 149/150 | G,R,I,S,H,D,K,Y,E,C,T,L,V,N,A |
| 425 | N | ASN425:A | 1.015 | 1 | 0.586, 1.182 | 3,1 | 148/150 | H,F,D,K,E,Y,G,R,S,Q,A,P,L,T,N |
| 426 | K | LYS426:A | 1.555 | 1 | 0.838, 1.711 | 2,1 | 149/150 | N,V,T,P,A,Q,S,G,I,R,E,K,D,H |
| 427 | E | GLU427:A | 0.183 | 4 | -0.051, 0.384 | 5,4 | 148/150 | P,T,N,Q,G,S,D,E,K |
| 428 | E | GLU428:A | -0.725 | 8 | -0.861,-0.640 | 8,7 | 147/150 | G,R,X,N,Q,D,E |
| 429 | K | LYS429:A | -0.087 | 5 | -0.349, 0.073 | 6,5 | 148/150 | I,R,M,K,E,L,T,V,A,Q |
| 430 | V | VAL430:A | -0.783 | 8 | -0.908,-0.701 | 8,7 | 148/150 | L,V,I |
| 431 | V | VAL431:A | -0.400 | 6 | -0.576,-0.260 | 7,6 | 148/150 | L,V,I,Y |
| 432 | G | GLY432:A | -1.261 | 9 | -1.302,-1.239 | 9,9 | 148/150 | G |
| 433 | I | ILE433:A | 0.231 | 4 | -0.051, 0.384 | 5,4 | 148/150 | F,C,A,V,L,I |
| 434 | H | HIS434:A | -1.237 | 9 | -1.291,-1.210 | 9,9 | 148/150 | Q,H,G |
| 435 | M | MET435:A | -0.414 | 6 | -0.576,-0.349 | 7,6 | 148/150 | V,L,T,G,M,I,F,Y,C |
| 436 | Q | GLN436:A | -0.223 | 6 | -0.431,-0.161 | 7,6 | 148/150 | C,M,I,A,Q,T,V,L |
| 437 | G | GLY437:A | -0.974 | 8 | -1.111,-0.908 | 9,8 | 148/150 | A,D,S,M,G |
| 438 | L | LEU438:A | 1.243 | 1 | 0.838, 1.711 | 2,1 | 148/150 | A,Q,N,P,T,L,V,Y,E,D,F,H,R,G,M |
| 439 | G | GLY439:A | 0.069 | 5 | -0.161, 0.216 | 6,4 | 148/150 | H,D,E,G,S,A,V,T,N |
| 440 | C | CYS440:A | -0.836 | 8 | -0.953,-0.757 | 8,8 | 148/150 | C,A,V,G,M,S |
| 441 | D | ASP441:A | -0.477 | 7 | -0.640,-0.349 | 7,6 | 148/150 | S,G,P,A,D |
| 442 | E | GLU442:A | -1.269 | 9 | -1.302,-1.266 | 9,9 | 148/150 | E,D |
| 443 | M | MET443:A | -0.688 | 7 | -0.811,-0.640 | 8,7 | 148/150 | V,L,I,M |
| 444 | L | LEU444:A | -0.370 | 6 | -0.576,-0.260 | 7,6 | 148/150 | S,I,G,M,A,V,T,L |
| 445 | Q | GLN445:A | -1.285 | 9 | -1.302,-1.291 | 9,9 | 148/150 | Q |
| 446 | G | GLY446:A | -0.153 | 6 | -0.431,-0.051 | 7,5 | 148/150 | A,T,L,V,C,S,G,M,I |
| 447 | F | PHE447:A | 0.101 | 5 | -0.161, 0.216 | 6,4 | 148/150 | F,Y,A,L,V,M,I |
| 448 | A | ALA448:A | -0.644 | 7 | -0.811,-0.576 | 8,7 | 148/150 | S,G,A |
| 449 | V | VAL449:A | -0.670 | 7 | -0.811,-0.576 | 8,7 | 148/150 | A,I,V,L |
| 450 | A | ALA450:A | -1.010 | 9 | -1.111,-0.953 | 9,8 | 148/150 | A,C,S,G,P |
| 451 | V | VAL451:A | 0.444 | 3 | 0.073, 0.586 | 5,3 | 148/150 | F,L,V,M,I |
| 452 | K | LYS452:A | -0.468 | 7 | -0.640,-0.349 | 7,6 | 148/150 | Q,T,V,N,H,K,G,M,R,S |
| 453 | M | MET453:A | -0.656 | 7 | -0.811,-0.576 | 8,7 | 148/150 | L,V,N,Q,A,G,M,S,C,K |
| 454 | G | GLY454:A | -0.761 | 8 | -0.908,-0.640 | 8,7 | 148/150 | N,R,G,E,K |
| 455 | A | ALA455:A | -0.670 | 7 | -0.811,-0.576 | 8,7 | 148/150 | L,V,I,C,A,F |
| 456 | T | THR456:A | -0.992 | 8 | -1.074,-0.953 | 9,8 | 148/150 | R,T,S,K,Y,C |
| 457 | K | LYS457:A | -1.046 | 9 | -1.146,-0.996 | 9,8 | 148/150 | A,T,V,L,F,K,Y,R,I |
| 458 | A | ALA458:A | 0.079 | 5 | -0.161, 0.216 | 6,4 | 148/150 | K,E,C,H,D,S,R,G,A,Q,N,T |
| 459 | D | ASP459:A | -0.104 | 5 | -0.349, 0.073 | 6,5 | 148/150 | E,D,H,M,I,A,Q,L,T,V |
| 460 | F | PHE460:A | -0.720 | 8 | -0.861,-0.640 | 8,7 | 148/150 | M,L,V,W,F |
| 461 | D | ASP461:A | -0.962 | 8 | -1.074,-0.908 | 9,8 | 148/150 | Q,V,L,N,D,H,E,C,K,M,R |
| 462 | N | ASN462:A | 0.877 | 2 | 0.384, 1.182 | 4,1 | 148/150 | S,R,K,E,D,N,L,T,A,Q |
| 463 | T | THR463:A | -0.978 | 8 | -1.074,-0.908 | 9,8 | 148/150 | S,L,V,T,A,C |
| 464 | V | VAL464:A | -0.211 | 6 | -0.431,-0.051 | 7,5 | 148/150 | I,M,T,V,L,F,C |
| 465 | A | ALA465:A | -1.039 | 9 | -1.146,-0.996 | 9,8 | 148/150 | P,G,A |
| 466 | I | ILE466:A | -0.900 | 8 | -0.996,-0.861 | 8,8 | 148/150 | I,V,L |
| 467 | H | HIS467:A | -1.280 | 9 | -1.302,-1.266 | 9,9 | 148/150 | H |
| 468 | P | PRO468:A | -1.268 | 9 | -1.302,-1.266 | 9,9 | 148/150 | P |
| 469 | T | THR469:A | -1.006 | 9 | -1.111,-0.953 | 9,8 | 148/150 | V,T,S,C |
| 470 | S | SER470:A | -0.261 | 6 | -0.431,-0.161 | 7,6 | 148/150 | S,G,I,M,C,T,V,L,A |
| 471 | S | SER471:A | -0.819 | 8 | -0.953,-0.757 | 8,8 | 148/150 | A,S,G |
| 472 | E | GLU472:A | -1.283 | 9 | -1.302,-1.291 | 9,9 | 148/150 | E |
| 473 | E | GLU473:A | -1.212 | 9 | -1.266,-1.178 | 9,9 | 147/150 | T,V,D,E |
| 474 | L | LEU474:A | -0.069 | 5 | -0.349, 0.073 | 6,5 | 147/150 | W,V,L,M,I,F |
| 475 | V | VAL475:A | -1.131 | 9 | -1.210,-1.111 | 9,9 | 144/150 | L,T,V,M,F,A |
| 476 | T | THR476:A | -1.000 | 9 | -1.111,-0.953 | 9,8 | 143/150 | K,S,R,I,L,P,T |
| 477 | L | LEU477:A | -0.476 | 7 | -0.640,-0.349 | 7,6 | 138/150 | L,I,M,F |
| 478 | R | ARG478:A | -0.979 | 8 | -1.074,-0.908 | 9,8 | 105/150 | K,R |

- POS: The position of the AA in the SEQRES derived seqence.

- SEQ: The SEQRES derived sequence in one letter code.

- 3LATOM: The ATOM derived sequence in three letter code, including the AA's positions as they appear in the PDB file and the chain identifier.

- SCORE: The normalized conservation scores.

- COLOR: The color scale representing the conservation scores (9 - conserved, 1 - variable).

- CONFIDENCE INTERVAL: When using the bayesian method for calculating rates, a confidence interval is assigned to each of the inferred evolutionary conservation scores.

- CONFIDENCE INTERVAL COLORS: When using the bayesian method for calculating rates. The color scale representing the lower and upper bounds of the confidence interval.

- MSA DATA: The number of aligned sequences having an amino acid (non-gapped) from the overall number of sequences at each position.

- RESIDUE VARIETY: The residues variety at each position of the multiple sequence alignment.

####
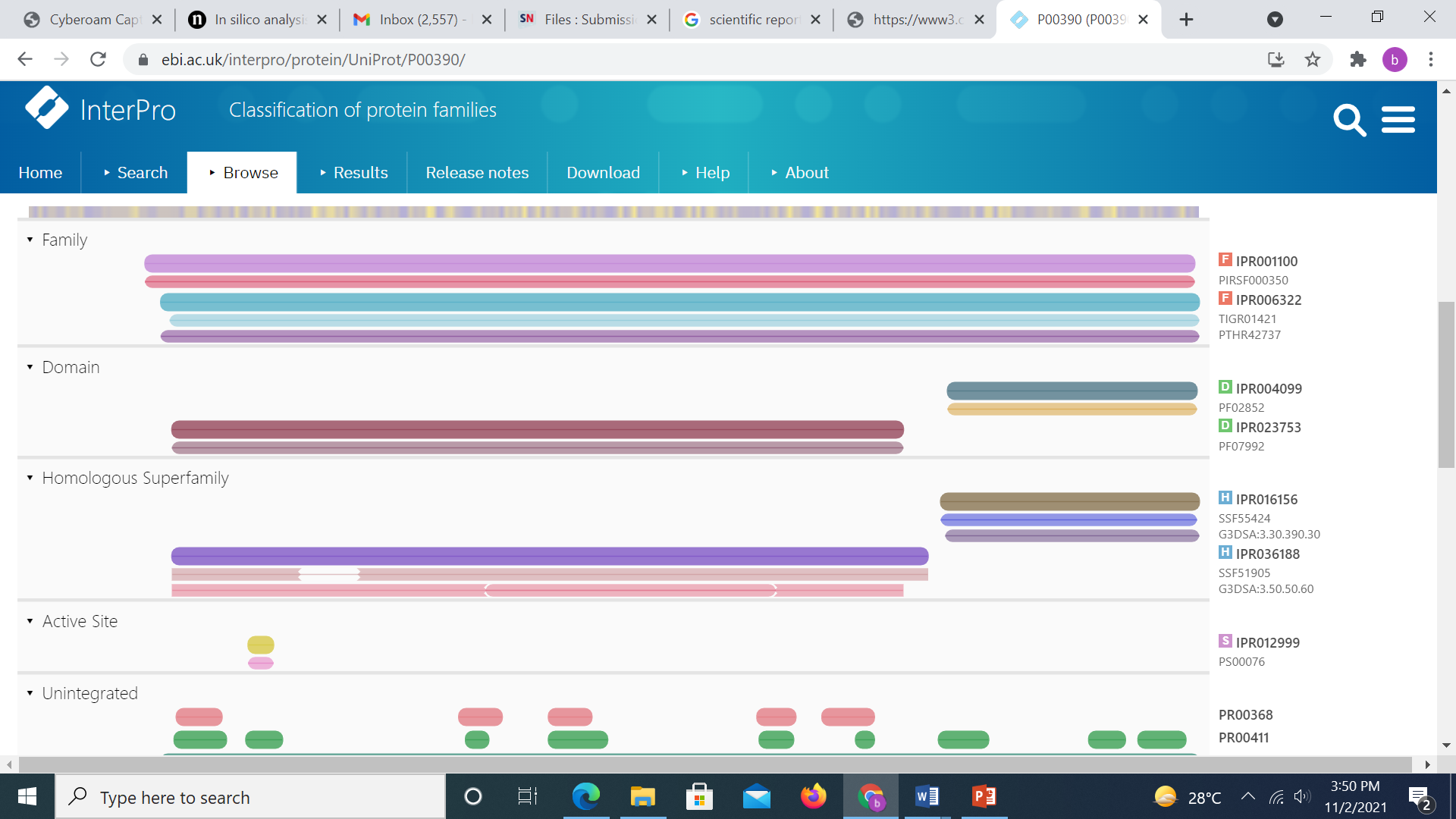


#### Figure S1: Domain identification of GSR protein using InterPRO server.

#### IPR006322 indicates the GSR protein (65-522aa), [IPR00](https://www.ebi.ac.uk/interpro/entry/InterPro/IPR002219/)4099 indicates Pyridine nucleotide-disulphide oxidoreductase domain, indicates and FAD/NAD(P)-binding domain (411-521), IPR023753 (65-39) indicates FAD/NAD(P)-binding domain.

**Table S2: Structural effect of 11 nsSNPs over RASSF5 protein using Project Hope**

| **Residue** | **Structure** | **Properties** |
| --- | --- | --- |
| R153C | 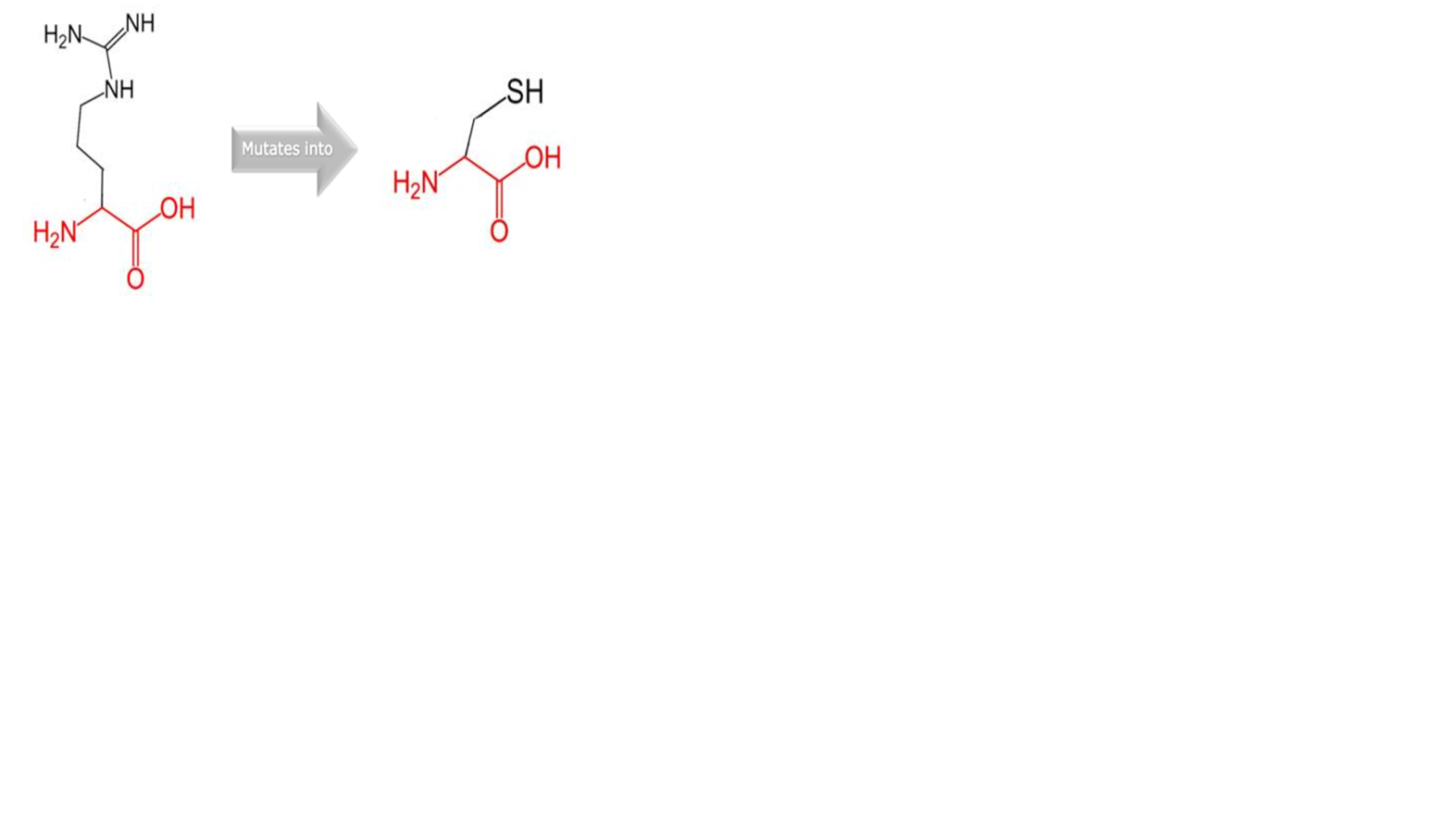 | The mutant residue is smaller than the wild-type residue.  The wild-type residue charge was POSITIVE, the mutant residue charge is NEUTRAL.  The mutant residue is more hydrophobic than the wild-type residue. |
| A199T | 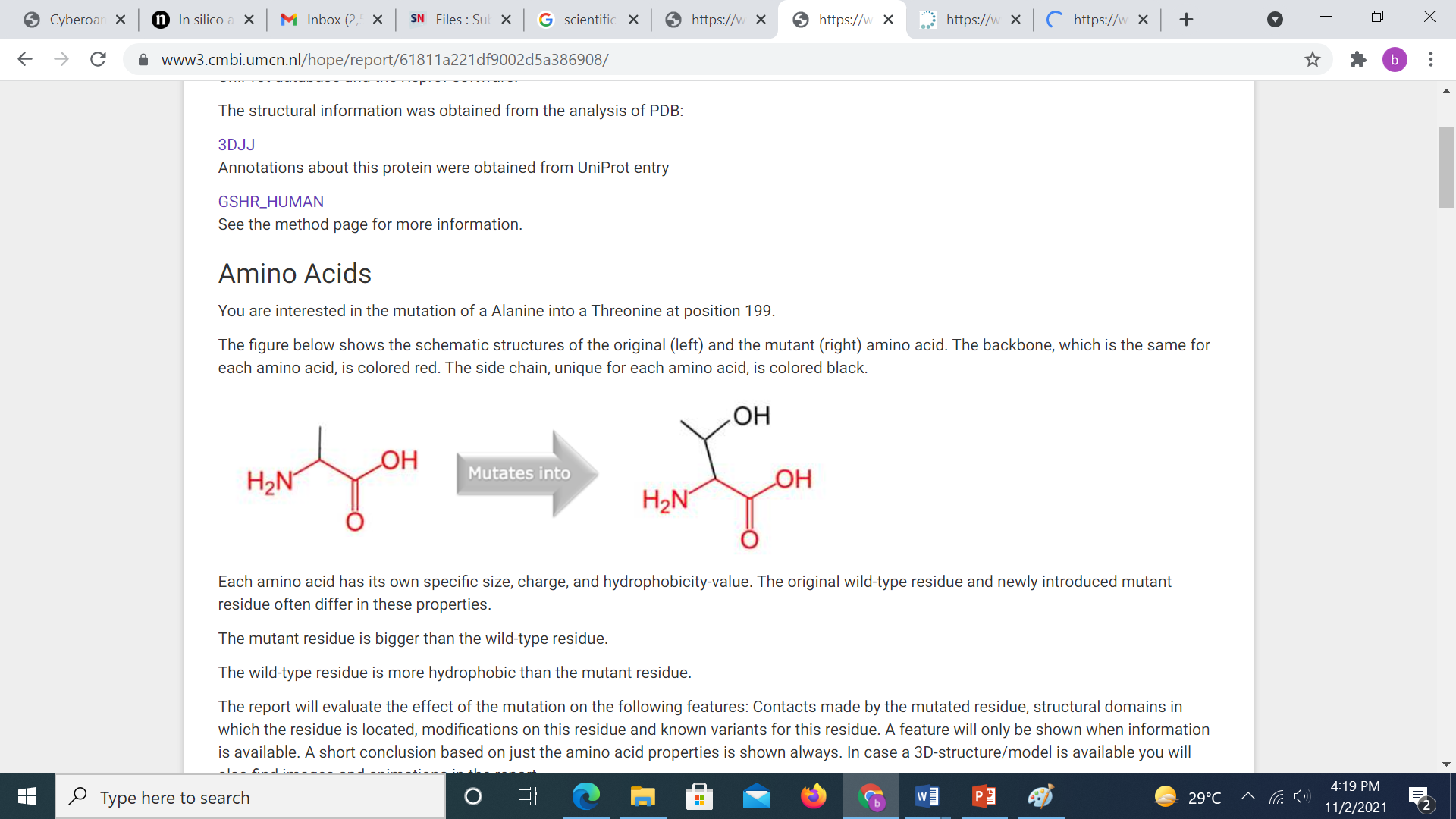 | The mutant residue is bigger than the wild-type residue.  The wild-type residue is more hydrophobic than the mutant residue. |
| R233C | 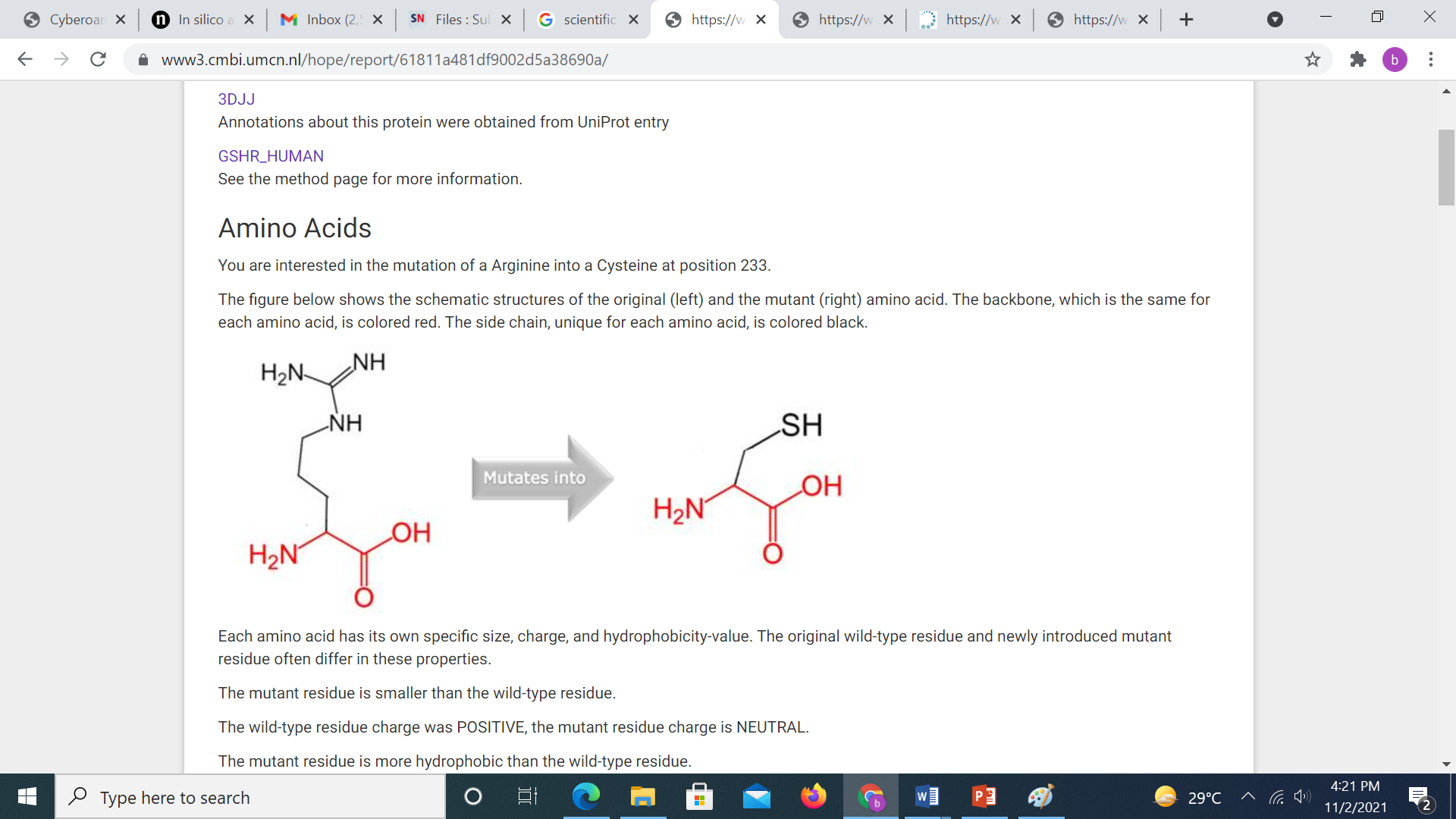 | The mutant residue is smaller than the wild-type residue.  The wild-type residue charge was POSITIVE, the mutant residue charge is NEUTRAL.  The mutant residue is more hydrophobic than the wild-type residue. |
| V298A | 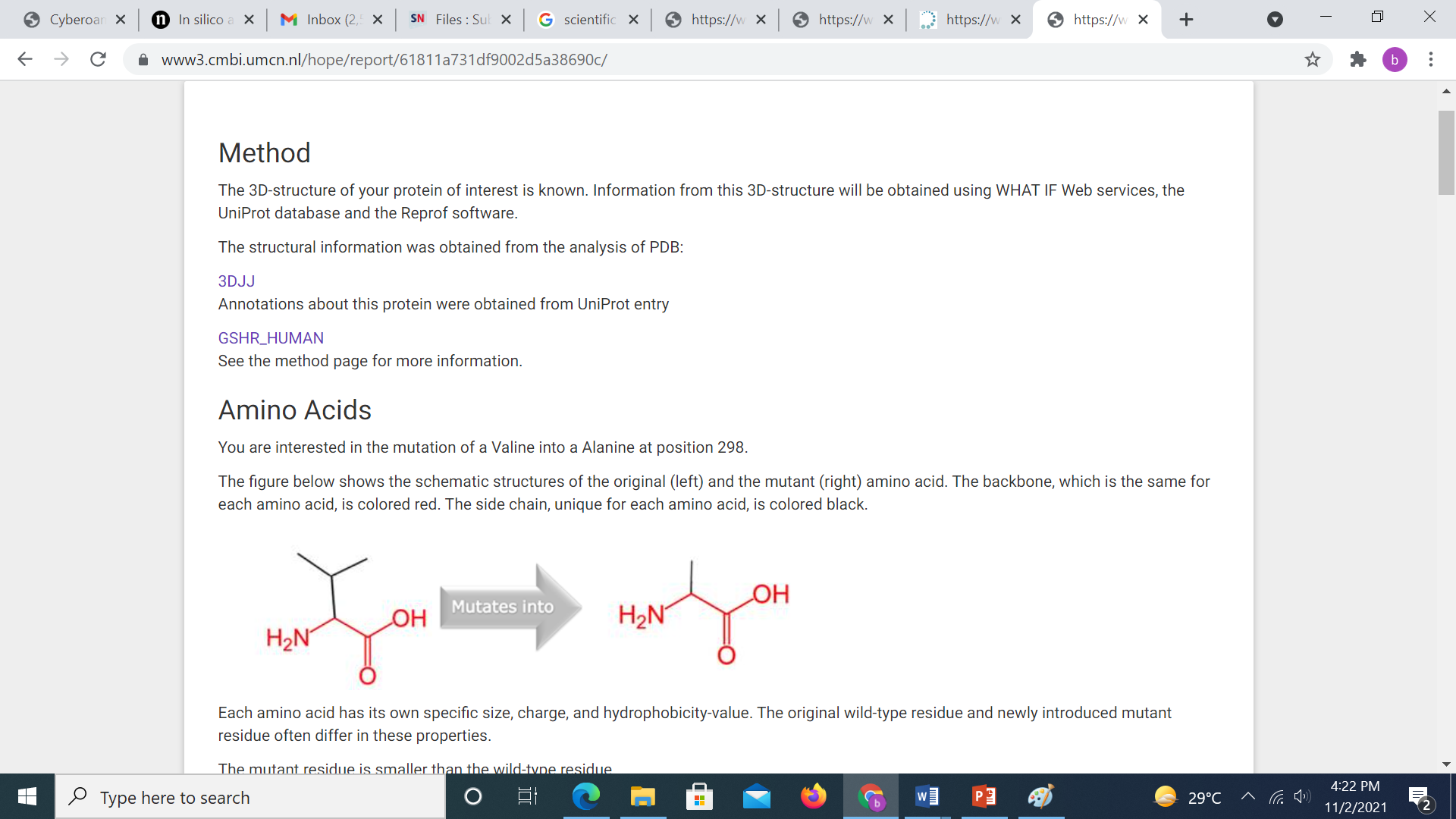 | The mutant residue is smaller than the wild-type residue. |

**TableS3: The significant SNPs in GSR gene with their allele information.**

| S. No. | SNPs | Position | ObsHET | PredHET | HWpval | %Geno | FamTrio | MendErr | MAF | Alleles | Rating |
| --- | --- | --- | --- | --- | --- | --- | --- | --- | --- | --- | --- |
| 1 | rs2738981 | 30618643 | 0.0 | 0.0 | 1.0 | 100.0 | 0 | 0 | 0.0 | C:C | BAD |
| 2 | rs8190968 | 30619193 | 0.0 | 0.0 | 1.0 | 100.0 | 0 | 0 | 0.0 | C:C | BAD |
| 3 | rs3757917 | 30619688 | 0.089 | 0.085 | 1.0 | 100.0 | 0 | 0 | 0.044 | C:T |  |
| 4 | rs8190966 | 30619734 | 0.0 | 0.0 | 1.0 | 100.0 | 0 | 0 | 0.0 | A:A | BAD |
| 5 | rs3757918 | 30619789 | 0.533 | 0.498 | 0.917 | 100.0 | 0 | 0 | 0.467 | T:C |  |
| 6 | rs2738976 | 30620943 | 0.0 | 0.0 | 1.0 | 95.6 | 0 | 0 | 0.0 | T:T | BAD |
| 7 | rs2551709 | 30621892 | 0.0 | 0.0 | 1.0 | 100.0 | 0 | 0 | 0.0 | T:T | BAD |
| 8 | rs8190955 | 30623292 | 0.022 | 0.022 | 1.0 | 100.0 | 0 | 0 | 0.011 | C:T |  |
| 9 | rs2551707 | 30623552 | 0.0 | 0.0 | 1.0 | 100.0 | 0 | 0 | 0.0 | C:C | BAD |
| 10 | rs2978663 | 30623613 | 0.489 | 0.488 | 1.0 | 100.0 | 0 | 0 | 0.422 | A:G |  |
| 11 | rs8190942 | 30624885 | 0.0 | 0.0 | 1.0 | 93.3 | 0 | 0 | 0.0 | C:C | BAD |
| 12 | rs8190939 | 30626100 | 0.0 | 0.0 | 1.0 | 100.0 | 0 | 0 | 0.0 | G:G | BAD |
| 13 | rs2551703 | 30626994 | 0.0 | 0.0 | 1.0 | 100.0 | 0 | 0 | 0.0 | C:C | BAD |
| 14 | rs2978662 | 30627495 | 0.289 | 0.278 | 1.0 | 100.0 | 0 | 0 | 0.167 | T:C |  |
| 15 | rs7832572 | 30630158 | 0.0 | 0.0 | 1.0 | 100.0 | 0 | 0 | 0.0 | T:T | BAD |
